# Review of Passive Shielding Materials for High-Energy Charged Particles in Earth’s Orbit

**DOI:** 10.3390/ma18112558

**Published:** 2025-05-29

**Authors:** Mingxin Wang, Qian Wang, Yakai Xiao, Mingliang Wang, Jianwei Wang, Haowei Wang, Zhansheng Chen

**Affiliations:** 1 State Key Laboratory of Metal Matrix Composites, Shanghai Jiao Tong University, Shanghai 200240, China; 123050910146@sjtu.edu.cn (M.W.); hwwang@sjtu.edu.cn (H.W.); 2 Shanghai Institute of Satellite Engineering, No. 3666 Yuanjiang Road, Shanghai 201109, China; wangqian_322@163.com (Q.W.); yakai_xiao@163.com (Y.X.); zhangsr@hanlly.com (Z.C.); 3 Institute of Alumics Materials, Shanghai Jiao Tong University (Anhui), Huaibei 235000, China

**Keywords:** Earth orbit, passive shielding technology, proton shielding material, electron shielding material, multi-layer shielding, advanced materials

## Abstract

As space missions become increasingly complex, protection against high-energy charged particles has emerged as a critical factor for the safe operation of spacecraft. These electrical particles, including protons and electrons, can penetrate spacecraft structures and cause severe damage to internal components. Therefore, this review discusses the characteristics of the high-energy charged particle environment in Earth orbits. Accordingly, various passive shielding materials have been evaluated, highlighting their advantages, disadvantages, and applicability in different orbital environments. Specifically, the importance of optimizing shielding materials and structures to enhance the radiation resistance of spacecraft has been emphasized. Furthermore, advancements in passive shielding materials for high-energy charged particles in Earth orbit over the past few years have been examined. Finally, future research directions have been proposed, including the development of lighter and more efficient shielding materials, the optimization of multi-layer shielding structures, and the integration of passive shielding with other protective technologies.

## 1. Introduction

With the continuous exploration of the universe by humanity, space technology has achieved remarkable advancements. In the early days when humans first ventured into space, this was driven by the pioneering efforts of space agencies (i.e., NASA and the former Soviet space program). Currently, ambitious deep-space exploration missions, the construction of complex space stations, and the rise of commercial space endeavors are spearheaded by companies (e.g., SpaceX and Blue Origin). Therefore, the field of space exploration has become an iconic symbol of human technological progress. These achievements not only showcase humanity’s ability to push the boundaries of science and engineering but also highlight the potential for future discoveries and innovations in space [1]. However, as the complexity of space missions increases, the space environment which spacecraft faces has become more challenging [2]. Among these challenges, protection against high-energy charged particles has gradually emerged as one of the key factors restricting the safe operation of spacecraft [3,4,5,6]. This issue is particularly critical given the growing duration and distance of modern space missions, exposing the spacecraft to prolonged periods of radiation [7,8].

High-energy charged particles, including protons, electrons, and heavy ions in cosmic rays, are ubiquitous in space [9]. These particles possess extremely high energy and strong penetrating power, enabling them to penetrate the outer shells of spacecraft. This can cause severe damage to satellite casings or internal optical/electronic components. For instance, high-energy protons and heavy ions can easily penetrate the satellite’s outer shell and directly impact the internal electronic components, leading to their performance degradation or even complete failure. The effects can range from temporary malfunctions, known as single-event upsets (SEUs), to permanent damage that renders the components inoperable [10]. Optical components are also vulnerable to the impact of high-energy particles. Exposure to these particles can result in surface damage or performance degradation, affecting the imaging quality and data transmission capabilities of satellites. This is particularly problematic for missions that rely on high-resolution imaging, such as Earth observation satellites or telescopes designed to capture detailed astronomical data. The degradation of optical components can lead to reduced image clarity, increased noise, and ultimately, compromised mission objectives [11,12,13,14].

In response to these challenges, satellite engineering has set higher requirements for radiation resistance. Radiation resistance refers to the ability of satellite materials to remain stable under long-term bombardment by high-energy particles. Not only is the in-depth study of materials that shield against high-energy charged particles for satellites of significant scientific importance, but also, it holds immense value for practical applications [15]. By leveraging passive protection technologies and developing high-performance shielding materials, it is possible to effectively extend the operational lifespan of satellites, enhance their reliability and safety, and provide a crucial safeguard for the success of space missions.

This article provides a comprehensive review of the literature on protection against high-energy charged particles in Earth’s orbital environment over the past few decades. It discusses various aspects, including the characteristics of the high-energy charged particle environment, the technologies and materials for passively shielding these particles, and the advantages, disadvantages, and applicable scenarios of different shielding techniques. By summarizing recent research findings and practical applications, this review aims to offer valuable insights for the design and implementation of effective shielding strategies to enhance the reliability and safety of satellites.

## 2. Environment of High-Energy Charged Particles in Earth’s Orbit

### 2.1. Classification of Earth’s Orbits

Commonly used Earth orbits include the low Earth orbit (LEO), medium Earth orbit (MEO), and geostationary Earth orbit (GEO) [16]. Typical features of these orbits include the following:

(1) The LEO extends from ≈400 km to 2000 km above the Earth’s surface. Numerous small satellites are deployed in this region to facilitate closer Earth observations and Remote Sens [17,18]. The South Atlantic Anomaly is a prominent feature in this area, characterized by elevated radiation levels due to the offset and tilt of the geomagnetic axis relative to Earth’s rotational axis [19]. In addition to the SAA, the polar horns also contribute to radiation analysis at the lower altitudes (e.g., orbits similar to the International Space Station). The polar horns are segments of the outer radiation belts that are in close proximity to Earth [20,21].
(2) The MEO spans an altitude range of 2000 km to 36,000 km. These orbits are situated near the center of Earth’s outer radiation belt, presenting a more intense radiation environment. This radiation can induce SEUs and latch-up phenomena in large-scale integrated electronic components, causing interference and introducing uncertain radiobiological effects [22,23,24].
(3) The GEO is located at an altitude of >36,000 km. Cosmic rays play a significant role in this orbit. Although the fluxes of these particles are relatively low, they include heavy and energetic ions (e.g., iron), producing intense ionization as they traverse matter. Shielding against these ions is challenging, making them a substantial hazard. Similarly to other orbits, they can trigger SEUs and latch-up in large-scale integrated electronic components to induce interference and uncertain radiobiological effects [25,26].

### 2.2. Radiation Environment in Earth’s Orbit

Generally, the radiation environment in Earth’s orbit is extremely complex, and high-energy charged particles in medium–high Earth orbits mainly come from galactic cosmic rays, solar cosmic rays, and Earth’s radiation belts, as shown in Table 1 [27].

**Galactic cosmic rays** are high-energy charged particles produced by supernova explosions. They primarily originate from celestial bodies within the Milky Way, excluding the Sun. Protons account for ≈85% of galactic cosmic rays as the dominant component [28]. He nuclei (α particles) make up ≈12% as the second-most significant component. Heavy-nuclei ions, including those composed of the nuclei of elements such as Li, Be, and B, constitute ≈1% of galactic cosmic rays. Electrons represent a smaller proportion of galactic cosmic rays but have a broader energy distribution range [29,30,31,32,33,34]. Given that heavy ions constitute a relatively small fraction of galactic cosmic rays, we focus primarily on higher-energy protons and high-energy electrons, which are more abundant.

The energy range of galactic cosmic rays is extremely wide, spanning from a few MeV to over 10^14^ MeV. The energy distribution of galactic cosmic rays is characterized by the following features: (1) Particles in the low-energy range are more common; (2) particles in the high-energy range are less frequent but can reach extremely high levels (Even >10^14^ MeV). Generally, the energy spectrum of galactic cosmic rays is “hard”, meaning that high-energy particles are relatively abundant, whereas low-energy particles are relatively scarce [35,36]. The intensity and distribution of galactic cosmic rays are significantly influenced by solar activity. Due to their abundance of high-energy particles and broad energy distribution, galactic cosmic rays pose a long-term radiation risk to spacecraft and astronauts in deep-space exploration missions [37,38,39].

**Solar cosmic rays** are high-energy charged particle streams emitted during solar eruptions. They are primarily composed of protons (accounting for ≈90%), along with a small number of electrons (≈9%), He nuclei, and heavy-nuclei ions with an atomic number Z > 2 (≈1%). The energy of protons typically ranges from 10 MeV to 1 GeV, while electrons generally have energies below 100 MeV. The energy spectrum of solar cosmic rays is “softer” compared to that of galactic cosmic rays, meaning that low-energy particles are more abundant, whereas high-energy particles are relatively scarce [40]. The solar activity cycle significantly affects the intensity and distribution of solar cosmic rays. During periods of high solar activity, the intensity of solar cosmic rays increases markedly, placing greater stress on the radiation environment for satellites [41,42].

**Figure 1:**
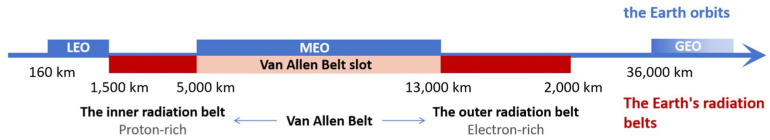
The correlation between the Van Allen Belts and satellite orbits.

**Earth’s radiation belts** in Earth’s orbit can be divided into the inner radiation belt and the outer radiation belt based on their altitudes. Its position relative to Earth’s orbit is depicted in Figure 1.

(1) The inner radiation belt is located at an altitude of ≈1000 km to 6000 km above the Earth’s surface, with a magnetic shell number (L) ranging from about 1.2 to 2. It is primarily composed of high-energy protons with energies ranging from 10 MeV to 100 MeV. Additionally, the inner radiation belt also contains lower-energy electrons, typically in the range of 10 keV to 100 keV. The inner radiation belt is relatively stable, with its particle distribution and intensity being less affected by solar activity and geomagnetic activity [43].
(2) The outer radiation belt is situated at an altitude of ≈13,000 km to 60,000 km above the Earth’s surface, with a magnetic shell number (L) ranging from about 3 to 8. It is mainly composed of high-energy electrons with energies ranging from 1 MeV to 10 MeV. The outer radiation belt also contains a small number of protons, but their energies and distribution differ from those in the inner radiation belt. The particle flux and distribution in the outer radiation belt are highly dynamic and significantly influenced by space weather events (i.e., geomagnetic storms and substorms) [44,45,46,47,48]. For example, during geomagnetic storms, the flux of high-energy electrons in the outer radiation belt can vary by several orders of magnitude within a few hours to a few days.

Between the higher boundary of the inner radiation belt and the lower boundary of the outer radiation belt, there is the Van Allen Belt slot, where there are mainly some low-energy particles, e.g., low-energy electrons. Typically, the Van Allen Belt slot is a relatively stable region whose particle density and distribution states remain relatively stable for longer time periods.

The LEO is below the inner radiation belt. The MEO is situated between the inner and outer radiation belts (also known as the Van Allen Belt slot). The GEO is above the outer radiation belt. All three orbits are susceptible to the influence of Earth’s radiation belts (especially during geomagnetic storms).

### 2.3. Negative Impacts of the Radiation Environment on Normal Operation of Satellites

High-energy charged particles from different radiation belts have extremely serious impacts on the normal operation of satellites, mainly manifesting in the following aspects [49,50]:

(1) **Displacement Effects:** High-energy particles can create lattice defects in the functional parts of devices when they enter. These lattice defects can lead to the degradation of device performance parameters (such as the conversion efficiency of photoelectric-sensitive devices) and gradual loss of functionality [51]. For example, when high-energy protons hit Si-based semiconductor materials, they can create defects in the lattice, reducing the material’s electrical performance.
(2) **Ionization Effects:** When charged particles enter electronic components, they ionize the bound electrons through an ionization process, generating a large number of electron–hole pairs. The ionization effect has little impact on metals, because the electronic states of electrons and holes in the conduction band of metals are already abundant. The increase in numbers due to ionization is insufficient to cause changes in their electrical properties. For semiconductors and insulators, the transition of electrons from the valence band to the conduction band should affect their electrical, chemical, and physical–mechanical properties. These electron–hole pairs can interfere with the normal operation of electronic components, leading to increased signal noise, performance degradation, and even complete failure [52,53,54]. For example, in semiconductor devices, ionization effects can increase the leakage current and reduce the switching performance of devices.
(3) **Single-Event Effects:** The single-event effect (SEE) refers to the phenomenon where the state of a microelectronic device undergoes an abnormal change due to ionization or nuclear reactions when a single high-energy particle (e.g., the proton, heavy ion, or electron) passes through the sensitive region of the device. High-energy particles can generate mobile charges in sensitive areas of devices, causing logic errors, latch-up, voltage drift, and device burnout in digital integrated circuits. The types of single-event effects include the SEU, single-event latch-up (SEL), single-event burnout (SEB), and single-event gate rupture (SEGR). SEEs are one of the most common radiation damages in spacecraft electronic systems and can lead to sudden system crashes, data loss, or erroneous operations.
(4) **Charging/Discharging Effects:** Charged particles can accumulate on the surface of spacecraft, forming high electric potentials. When the potential difference between different parts of a charged spacecraft exceeds a critical value, discharge phenomena can occur. The discharge process can release charges, heat, electromagnetic pulses, and glow, potentially altering the performance of the materials at the discharge site. Moreover, charges and electromagnetic pulses can directly or indirectly enter the electronic circuits or electrical systems of spacecraft, causing fatal damage to their safe operation.
(5) Statistics show that up to 70% of in-orbit satellite failures are induced by the high-energy particle radiation environment in space [55,56]. For example, China’s “Fengyun-1B” meteorological satellite experienced multiple SEUs caused by high-energy particles in space after 165 days in orbit, leading to sudden failures of the onboard computer and premature failure. Additionally, the space radiation environment is closely related to the solar activity cycle. In 2003, frequent solar activity led to numerous in-orbit anomalies in spacecraft. Among these anomalies, most were temporary and self-recoverable. For instance, the US “Chandra” X-ray Observatory experienced an anomaly on 24 October 2003, but resumed normal operation on the 25th. However, some spacecraft suffered permanent failures. For example, Japan’s “Adeos2” satellite entered safe mode due to radiation effects, and it was eventually scrapped due to power supply failure. The impact of space radiation effects caused the “Adeos2” satellite to operate for only 10 months in orbit, which was three years less than its planned lifespan.
(6) Therefore, how to protect against radiation is an important issue in satellite engineering, and the study and use of materials with high shielding capabilities are key to ensuring the normal service of satellites.

### 2.4. Radiation Protection Measures

In aerospace engineering, protecting against high-energy charged particles is an important topic. The main protection methods include electric field shielding, magnetic field shielding, fault-tolerant computing, protective circuits, encapsulation shielding of electronic components, and mass shielding of specific areas [57,58]:

(1) **Electric Field Shielding:** This involves using electric fields to generate a bias that prevents or deflects the motion of charged particles. By applying an appropriate electric field to the surface of a spacecraft, the trajectory of charged particles can be altered to divert them away from critical components of the spacecraft.
(2) **Magnetic Field Shielding:** This involves using magnetic fields to change the direction of incoming particles to provide shielding. Magnetic field shielding can be achieved by placing a magnetic field generator around the spacecraft, thereby deflecting high-energy charged particles and reducing their direct impact on the spacecraft.
(3) **Fault-Tolerant Computing:** This includes dual-system configurations, also known as computer fault-tolerant technology. By incorporating fault tolerance and error correction capabilities, computers can continue to operate normally when errors occur or remain unaffected by errors for a certain period of time.
(4) **Protective Circuitry:** This involves using ground-based remote control or an automatic system protection mechanism to cut off power supply. The system can then resume operation once the space environment improves. This method can protect electronics from damage when the radiation environment deteriorates.
(5) **Electronic Component Packaging Shielding:** This involves encapsulating electronic components with specialized materials or structures to protect them from external electromagnetic interference, radiation, and environmental factors, while also preventing the emission of electromagnetic signals from the components themselves.
(6) **Special Location Mass Shielding:** This refers to the application of targeted shielding measures to specific areas or components that are vulnerable to electromagnetic interference or radiation, ensuring the integrity and effectiveness of the overall shielding system.

Among these methods, passive protection is most widely applied due to its simplicity, reliability, and cost-effectiveness. It relies on the use of materials and structures which can absorb, reflect, or block harmful radiation or electromagnetic fields without requiring an external power source. In the following sections, we will delve into the principles, applications, and advancements of passive shielding materials, exploring how they can be optimized to enhance the safety and performance of various systems and environments.

### 2.5. Monte Carlo Simulations

The Monte Carlo simulation method is a fundamental approach used in particle simulation software. It can provide results in the form of probability distributions, which helps analyze uncertainties in complex systems. The accuracy of this method depends on the quality of the input data and the rationality of the assumptions. When the input data are accurate and the assumptions are reasonable, it can yield fairly reliable estimates. However, this simulation method has several drawbacks, including high computational intensity, heavy reliance on input assumptions and parameters, complex model structure, and high data requirements. Additionally, the accuracy of simulation results usually needs to be verified by comparison with actual data.

GEANT4 (Version 11.1.1) and MCNP (Monte Carlo N Particle Transport Code, Version 6.2) are software packages based on the Monte Carlo method. They are considered highly reliable for simulating radiation transport problems and are capable of providing more accurate results than discrete ordinates methods [59].

MULASSIS (Version v1.27) is a Geant4-based M-C simulation tool for dose and particle fluence analysis associated with the use of radiation shields. Users can define the shielding and detector geometry as planar or spherical layers, with the material in each layer defined by its density and elemental/isotopic composition. Incident particles can be any Geant4 particles, including protons, neutrons, electrons, gammas, alphas, and light ions. Users can choose from a wide range of energy levels and angular distributions. In addition, radiation spectra produced by SPENVIS (Version v4.6.12) can be input when the tool is used within this system.

## 3. Passive Shielding Materials

In-depth research into materials which can effectively shield against high-energy charged particles is of great scientific significance and immense practical application value. By developing high-performance shielding materials, the lifespan of satellites can be significantly extended. This not only reduces the cost of space missions by minimizing the need for frequent replacements but also enhances the reliability and safety of spacecraft, providing critical guarantees for the success of space missions [60].

Moreover, advanced shielding materials can enable more ambitious and longer-duration missions. For example, deep-space exploration missions to Mars or beyond require spacecraft to withstand prolonged exposure to cosmic radiation. Effective shielding materials can protect astronauts and onboard systems from harmful radiation, ensuring the safety and functionality of the spacecraft throughout the journey.

This section discusses passive shielding materials, which work best against the lowest-energy particles that do not produce secondaries and are most commonly used in the aerospace industry, covering proton shielding materials, electron shielding materials, and materials that shield against both.

### 3.1. Proton Shielding Materials

The design of proton shielding materials focuses on low-atomic-number (low-Z) materials. Correspondingly, the shielding mechanism involves interactions with protons through ionization and scattering. When high-energy charged protons interact with matter, they primarily lose energy through inelastic collisions with electrons outside the atomic nucleus. Low-Z materials have a lower average atomic mass, but a higher electron density per unit mass. This means that under the same areal density, low-Z materials can provide more free electrons, thereby more effectively interacting inelastically with the incident protons and reducing their energies. High-atomic-number (high-Z) materials can also shield against protons in some cases, but they produce more secondary radiation (such as bremsstrahlung and recoil protons) [61].

Novikov et al. [62] conducted a detailed study on the energy spectra of primary and secondary particles inside the Columbus laboratory module on the International Space Station. The results showed that although the spacecraft’s outer shell provided some shielding against incident particles, it also generated a large number of secondary particles. In particular, for protons in galactic cosmic rays, the proton flux inside the laboratory module was higher than that of the incident particles. This finding indicated that while the spacecraft’s outer shell can shield incident particles, it may also introduce additional radiation risks. Therefore, it is essential to consider the radiation effects of both primary and secondary particles when designing spacecraft structures to shield against high-energy charged protons.

#### 3.1.1. Traditional Proton Shielding Materials

**Figure 2:**
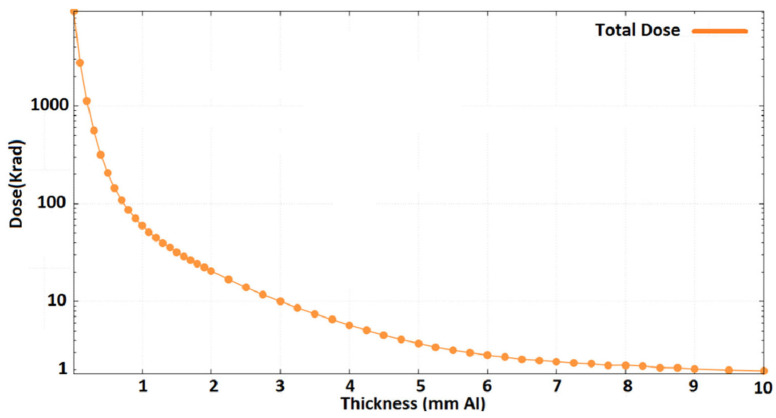
Dose–depth curve for the Al shielding layer in a satellite orbiting in LEO during a 3-year operation [63].

Traditional proton shielding materials often use common low-Z materials, including the following:

(1) **Polyethylene:** Polyethylene has a high content of low-Z elements (C and H), effectively slowing down protons and reducing their radiation damage. Furthermore, polyethylene has a low density, is easy to process, and has good mechanical properties (i.e., flexibility and impact resistance). Therefore, it is extensively utilized to fabricate shielding structures of various shapes and sizes, making it adaptable to intricate spatial arrangements. Meanwhile, the high impact resistance and good mechanical properties of polyethylene enable it to endure mechanical impacts and vibrations during operation, guaranteeing the stability of shielding effectiveness.
(2) **Al:** Al and alloys are easily processed and have relatively higher mechanical strength, meeting the higher structural shielding needs. For example, Sajid et al. [63] studied the shielding performance of Al layers, using the South Atlantic Anomaly as a typical case. They found that the maximum dose rate was significantly reduced from 10 rad/s to 0.01 rad/s after adding an Al shielding layer. Through Monte Carlo simulation, the protective performances of Al layers with different areal densities were evaluated. For a satellite orbit in LEO on a 3-year mission (Figure 2), the results indicated that a shield thickness of 3 mm attenuated the TID (Total Ionizing Dose) to less than 10 krad (Si). This is an acceptable total dose. To sum up, a 3 mm thick Al shielding layer meets the radiation shielding requirements for integrated devices with 65 nm and 130 nm processes. Current studies have suggested that Al is a lightweight and effective shielding material in LEO and MEO, which has significant application value in the protection of spacecraft against radiation.

However, these traditional shielding materials have drawbacks. For example, polyethylene has relatively lower high-temperature mechanical strength and poorer heat resistance, limiting its use in high-temperature environments. In comparison, Al has relatively weak proton shielding, limiting its effectiveness against high-energy protons.

#### 3.1.2. Advanced Proton Shielding Materials

**Figure 3:**
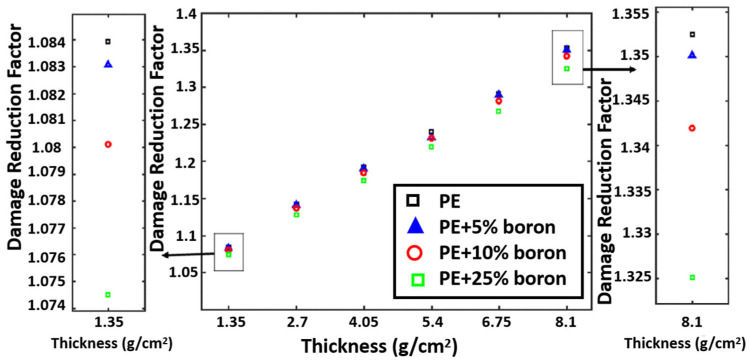
Damage reduction factor versus thickness for proton source [64].

Advanced proton shielding materials have been investigated in a variety of methods. For example, Vahedi et al. [64] developed effective proton shielding solutions for electronic components of LEO satellites, focusing on mitigating displacement damage caused by radiation. To achieve this, simulations were conducted using both the MCNP (Version 6.2) and OMERE (a toolkit for space environment, Version 5.9.1) software to model the radiation environment of LEO. Various shielding materials were tested, including polyethylene and borated polyethylene with different weight percentages of the B element. Their performances were compared to that of a standard Al shielding layer. The results showed that polyethylene and borated polyethylene shields significantly reduced displacement damage compared to Al. For proton shielding, a thickness of 8.10 g/cm^2^ of borated polyethylene achieved a 26% reduction in damage compared to the Al shield. In Figure 3, pure polyethylene was found to be effective in reducing proton-induced atomic displacement. For thicker shields, pure polyethylene was found to have higher damage reduction factors. Therefore, the choice of shielding material and its composition should be tailored based on the specific radiation environment and the required thickness of the shield. Their study highlighted the importance of material selection and composition optimization in designing effective radiation shields for electronic components in LEO satellites. The findings provide valuable insights for future satellite missions, where minimizing displacement damage is crucial for ensuring the reliability and longevity of onboard electronics.

With the development of materials science, a large number of new types of materials have emerged, driving the emergence and development of new shielding materials. In recent years, low-Z polymers have gained attention as new proton shielding materials. They retain the shielding principles of traditional low-Z materials and achieve performance breakthroughs through polymer properties. Among various shielding materials, those with low-Z materials, such as H, have been proven to be highly effective. Nevertheless, concerns over feasibility and safety have restricted their widespread use. One promising alternative is the use of H-rich polymer nanocomposites, which can offer effective radiation shielding with reductions in secondary radiation and weight [65,66]. Additionally, these nanocomposites enhance mechanical and physical properties, making them a viable replacement for metals [67]. For instance, the Ultra-High-Molecular-Weight Polyethylene Fiber (UHMWPEF), with a molecular weight of (1–5) × 10^6^ g/mol, is among the fibers with the highest specific strength and modulus. It offers excellent proton shielding and mechanical properties, making it a top choice for proton shielding. Additionally, these new polymer materials provide higher shielding efficiency and better flexibility, showing great promise in proton shielding.

Some studies have validated the shielding performance of nanocomposites through both ground and space experiments. For example, materials containing hydrogen, boron, and nitrogen (such as boron nitride nanotubes, BNNTs) have demonstrated superior shielding effectiveness compared to polyethylene in simulated space environments [68]. These materials not only excel at radiation shielding but also possess high structural strength, making them ideal shielding materials for future deep-space exploration missions.

### 3.2. Electron Shielding Materials

Electronic shielding materials rely on high-Z materials for their shielding effects. With high nuclear charges and many outer electrons, high-Z materials can effectively block electron penetration. When electron beams pass through, they interact with the atoms via the photoelectric effect and Compton scattering, transferring energy to the material’s electrons. This causes the incident electrons’ energy to decay until they can no longer penetrate the material, thereby providing protection. Thus, high-Z materials are crucial in electronic shielding, safeguarding people and equipment from electron radiation.

#### 3.2.1. Traditional Electron Shielding Materials

**Figure 4:**
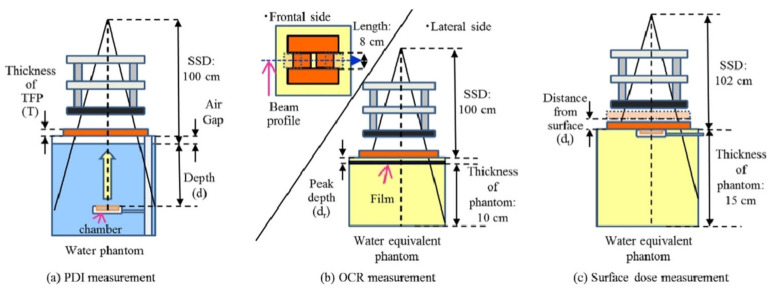
Schematic diagram showing the setup of the experiment: (**a**) percentage depth ionization measurement for 10 × 10 cm^2^ and 20 × 20 cm^2^ field sizes with different thicknesses of TFP (0.0, 1.5, 3.0, 4.5, 6.0, 12.0, and 15.0 mm), (**b**) off-center ratio measurement for 8 × 2, 10, 20 cm^2^ and 8 × 9 cm^2^ field sizes with 15 mm TFP or 3 mm Pb, and (**c**) surface dose measurement with the location of TFP being 0.0 cm and 2.5 cm from the phantom surface [71].

In the case of shielding against high-energy electrons, traditional materials commonly used include metallic materials:

(1) **Pb:** It is a metal commonly used for irradiation shielding, owing to its high density and atomic number. The high density allows it to absorb and scatter radiation effectively, reducing electron penetration. In addition, the large atomic number means better radiation shielding by the atomic nucleus, effectively blocking radiation. For example, Zhang et al. [69] prepared a Pb–B–polyethylene composite material by pre-treating and modifying B-containing compounds and then mixing them with Pb sand and polyethylene. They studied its radiation shielding. The results showed that at a density of 5.9 g/cm^3^ and thickness of 4.5 cm, the composite met the shielding requirements.
(2) **Fe:** Fe is also used for shielding against high-energy charged particle radiation as a typical engineering metal. Fujita et al. [70] investigated the types and doses of secondary radiation produced when high-energy electrons passed through an Fe shielding layer and found that a large number of bremsstrahlung X-rays and secondary neutrons were generated. Fe is a shielding material with certain shielding capabilities, but it is necessary to consider the generation of secondary particles and the potential impact on internal spacecraft components.
(3) **W:** W is a high-Z element and also a common shielding material. It is dense, hard, and corrosion-resistant, providing great high-energy radiation shielding capabilities. With the atomic number of 74, its atomic nucleus shields against radiation better than Pb, offering superior radiation attenuation in thinner layers. Also, its machinability allows it to be shaped into various forms and thicknesses, meeting diverse scenario needs. For example, Fujimoto et al. [71] developed a novel shielding material called Tungsten Functional Paper (TFP) through a unique fabrication process (Figure 4). This material was Pb-free, lightweight, flexible, and easily processed, containing up to 80% fine W powder by weight. They investigated the dosimetric changes and shielding performance of TFP for electron beams in radiotherapy. The TFP (with a thickness of 0–15 mm) was placed on water or a water-equivalent phantom to measure percentage depth ionization and transmission for 4, 6, and 9 MeV electron beams. Off-center ratios were also assessed using film dosimetry at the depth of dose maximum under similar conditions. Additionally, beam profiles and transmission were compared between TFP and Pb shielding materials. Their findings revealed that TFP achieved a 95% reduction in dose at 0.5 cm depth for 4, 6, and 9 MeV electron beams, respectively, at irradiation field sizes of 4, 9, and 15 mm. Notably, the dose tended to increase at the field edge shaped with TFP, which may be influenced by its thickness. The transmission of shielded electron beams was found to depend on the measurement depth and irradiation field size, with high radiation shielding effects observed based on the energy and TFP thickness used. Finally, they confirmed that W can be an effective material for electron shielding.

(4) **Ta:** As a high-Z element, Ta is a commonly used shielding material. It has a high melting point, high hardness, good corrosion resistance, and excellent conductivity. These properties make it excel in high-energy radiation shielding. Some scholars have studied Ta’s shielding performance. For example, Lasi et al. [72] investigated the electron shielding effect of Ta in the extreme radiation environment of Jupiter. The results showed that compared to a 6 mm thick Ta shield, an 8 mm thick Ta shield only increased the shielding effect by 30%. In contrast, the 6 mm thick Ta shielding material provided the optimal shielding effect in terms of both shielding performance and weight resources. This indicates that when designing radiation shielding schemes, it is necessary to balance shielding effectiveness and weight to meet the specific requirements of space missions.

Furthermore, NASA has also developed radiation-resistant vaults and shielding boxes using high-Z metal materials such as Ta and Ti. They have been successfully applied to the Juno Jupiter probe [73]. These studies demonstrate that high-Z metal materials have significant shielding advantages in extreme radiation environments and can effectively protect sensitive equipment inside spacecraft from damage caused by high-energy particles.

#### 3.2.2. Advanced Electron Shielding Materials

In addition to using pure metals for radiation protection, the addition of metal oxides to inorganic non-metallic materials has also been studied to improve shielding effectiveness [74]. One research direction for new electronic shielding materials is metal- and metal-oxide-doped nanoparticles. Using nanotechnology, these particles are evenly dispersed in a matrix to enhance traditional materials while maintaining shielding efficiency. For instance, metal-doped hollow silica nanoparticles are promising [75].

Their metal-doped shell offers a larger specific surface area and better mechanical properties, making them widely applicable in electronic shielding.

These novel materials, with their unique features and advantages, have great potential in electronic protection and can be used in medical devices, aerospace, and electronics, providing efficient electronic radiation shielding for people and equipment.

Finally, the mass efficiency, shielding efficiency, cost, manufacturability, and mechanical properties of proton shielding materials and electron shielding materials (Al, polyethylene, Pb, Fe, W, and Ta) are summarized in Table 2.

### 3.3. Materials for Shielding Against Protons and Electrons

To shield against both protons and electrons, the combination of both low-Z and high-Z materials is an effective solution. This composite material uses the synergy of low-Z and high-Z materials to block proton and electron penetration, offering more comprehensive protection.

#### 3.3.1. Shielding Composite Materials

A typical proton + electron shielding material combines polymers with metal/metal-oxide particles. It uses the polymer’s low-Z property to block protons and the metal/metal-oxide particles’ high-Z property to stop electrons. For example, polyethylene/Al composites have been found to excel in shielding [76]. Low-Z polyethylene shielded against protons effectively, while high-Z Al blocked electrons well. This combination not only outperforms single-material shields but also significantly reduces shield weight while meeting protection requirements. In satellite protection, especially for elliptical-orbit satellites passing through the inner radiation belt, the simulations showed that polyethylene/Al composite structures saved at least 27.8% of shielding mass compared to traditional Al-only structures, with the achievement of the same total-dose and displacement-dose protection goals [77].

Flexible silicon-based polymer/W composites are also important. For example, Srilakshmi et al. [78] tested the shielding efficiency of flexible Si-based polymer/W composites with different W contents and found that shielding performance increased with higher W content. The shielding efficiency at a given W content falls between that of Al and Fe. Furthermore, Brock et al. [79] developed new materials, such as lightweight composites impregnated with metallic nanoparticles, chitin-derived bioplastics, and aerogel-family materials, to offer advantages over current radiation shielding solutions. These composite materials show great potential in proton + electron shielding and can be widely used in medical devices, aerospace, and electronics, providing more efficient radiation protection.

#### 3.3.2. Multi-Layer Structured Materials

(1) **Single-layer structured shielding materials**
  A single-layer structured shielding material is a lightweight design that is particularly suitable for spacecraft with strict weight requirements. It is simple in structure and easy to install, effectively reducing the overall weight of the spacecraft, thereby lowering launch costs and increasing payload capacity. This design performs well in the radiation environment of both LEO and MEO, meeting basic radiation protection needs [80]. However, its shielding effectiveness against high-energy particles is limited. For example, in environments with both high-energy protons and electrons, its protective capability may be inferior to that of multi-layer structured material. Therefore, in complex radiation environments, such as transfer orbit missions passing through the inner radiation belt, single-layer shielding may not provide sufficient protection, and additional protective measures are needed. Overall, the single-layer structured shielding material has significant application value in scenarios that require lightweight design and have moderate radiation protection requirements [81].
(2) **Multi-layer structured materials**
  In addition to single-layer structured shielding materials, multi-layer structured shielding materials have also been studied and applied [73]. Without compromising structural strength and electrical conductivity, the protective performance of multi-layer shielding materials is superior to that of single-layer structured shielding materials by designing the types, thicknesses, and stacking order of materials [82,83]. Therefore, designing multi-layer shielding can optimize shielding effects [84].

For high-energy electron shielding, an outer low-Z layer slows electrons and cuts bremsstrahlung, while an inner high-Z layer blocks residual electrons [85,86]. For high-energy protons, an outer high-Z layer causes ionization energy loss, and an inner low-Z layer further slows and absorbs leftover energy [87,88]. In addition, to reduce secondary radiation, such as bremsstrahlung from high-Z materials, material selection can be optimized [89], the stacking order of materials can be adjusted, and the thickness of materials can be manipulated [90]. The optimal shielding effect can thereby be achieved. Also, several studies have been conducted to quantify secondary particle generation for different shielding materials. Theoretical calculations using Monte Carlo methods, such as the Particle and Heavy Ion Transport code System (PHITS), have been employed to quantify secondary particle generation. For example, polyethylene layers have been shown to reduce the absorbed dose rate of secondary particles significantly [91]. A multi-detector experimental setup has been used to measure secondary radiation behind thick shielding materials. This setup helps in assessing the effectiveness of different materials in mitigating secondary particle production [92].

In practice, multi-layer shielding technology is widely used in spacecraft and nuclear reactors to protect equipment and personnel from high-energy charged particle radiation. The designing principle is based on the different shielding capabilities of materials against protons and electrons. The order and thickness of each layer are carefully planned to achieve graded shielding against radiation of different energies and types. For example, in spacecraft radiation protection, the inner layer can be low-Z materials like polyethylene for proton shielding, while the outer layer can be high-Z materials like W or Ta for electron blocking [93]. The electric/magnetic dual-gradient composite film, developed with its excellent shielding performance and multifunctional characteristics, is expected to find applications in aerospace [94,95]. This layered design improves shielding efficiency and significantly reduces the weight of the shielding structure. Also, multi-layer structures are adaptable, allowing flexible adjustments to different radiation environments and protection requirements. They have shown excellent shielding performance in complex radiation environments [96].

Multi-layer shielding materials are categorized into three types: for proton shielding, electron shielding, or shielding against both, based on their shielding purposes.

➀ **Multi-layer materials in proton shielding**

Many studies have demonstrated that multi-layer materials are effective for proton shielding. For example, Daneshvar et al. [97] found that a three-layer structure of “Al bronze–molybdenum–Al bronze” can shield against proton radiation at an improved level over the traditional single-layer pure Al. In addition, these three-layer shielding materials offered greater processing versatility.

**Figure 5:**
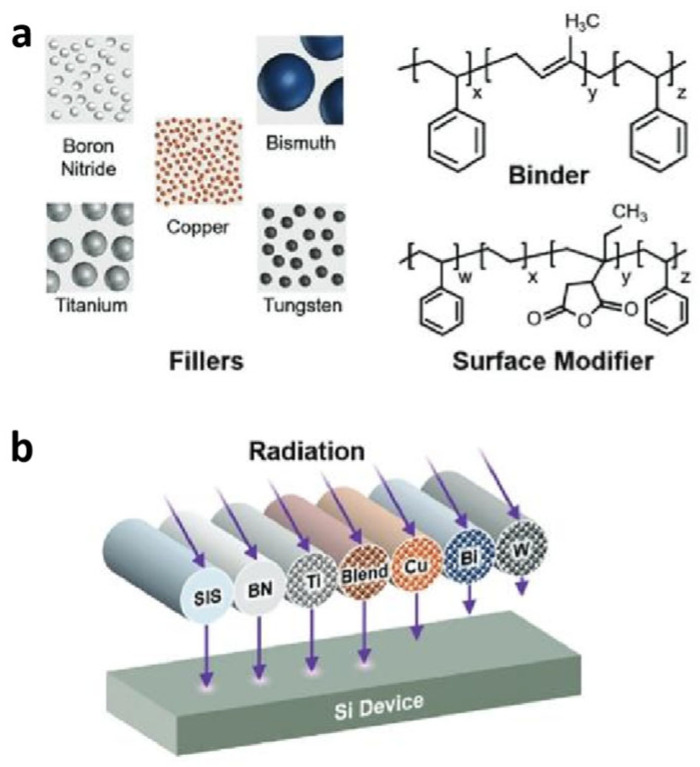
Three-dimensional printing of composites for radiation shielding. (**a**) Schematic depicting the components of the composite inks (In polymer chemistry, x, y, and z represent different positions in a chemical structure, typically used to mark the location of specific functional groups or substituents. This labeling method is commonly used to describe the functional or structural characteristics of different parts of a polymer chain. Specifically: x: Usually indicates a connecting point. y: Typically represents a middle section. z: Generally indicates another connecting point.). The filler, binder, and surface modifier are combined with toluene to form printable composites. (**b**) Schematic illustration of the printed filaments and their radiation attenuation behavior [98].

In addition, Avey et al. [98] described a versatile material platform and process that enabled the conformal printing of customized composite inks directly and selectively onto commercial, off-the-shelf electronics at room temperature (Figure 5). This modular platform leveraged a flexible styrene–isoprene–styrene (SIS) block copolymer binder, which can be filled with particles of varying atomic densities to achieve different radiation shielding capabilities. Additionally, the system allowed for the combination of multiple distinct particle species within a single printed structure, enabling the production of graded shielding that enhances radiation attenuation by tailoring both shield geometry and composition to provide comprehensive protection against a broad range of radiation types. These custom composite inks, with their tailored filler compositions, have been shown to outperform Al in attenuating protons. However, one limitation of this system is that the fabricated shields may underperform compared to pure high- and/or low-Z element layers, as the atomic density of the composite is the average of both the filler and the binder [98]. Despite this, the additive manufacturing approach offers significant advantages, including geometric freedom, minimal thermomechanical mismatches between dissimilar compositions, and precise control over material properties at the voxel level throughout the shield. Their future work should focus on translating the properties of the graded shields into three dimensions to further enhance the size, weight, power, and cost (SWaP-C) benefits of this approach.

Non-planar shield geometries with varying compositions can allow for a significant reduction in shield volume and can be readily fabricated via active mixing of inks during deposition. We also envision the incorporation of unique alloys and other multi-element particles into the composite, which should provide even greater control over the shield’s electromagnetic interactions. This method is anticipated to enable the rapid proliferation of the next generation of compact satellite designs by efficiently protecting state-of-the-art microelectronics with customized shields to their prospective radiation environment [98].

➁ **Multi-layer materials in electron shielding**

Multi-layer materials are also quite effective for electron shielding. For example, Borts et al. [65] investigated the impact of heterogeneity on the shielding effectiveness of laminated composites subjected to moderate-energy (≈MeV) electron irradiation. Their study quantified this effect using the Monte Carlo method, focusing on the directional dependence of Al-W shielding efficiency in a bimetallic dual-layer system, specifically comparing the Al-W and W-Al irradiation orientations. Simulations were conducted to model the transport of charged particles and bremsstrahlung radiation during electron irradiation of a nine-layer composite sample. This sample was fabricated through vacuum hot rolling solid-phase welding of Ti-Ni-Cu-Nb-Cu-(W-Cu)2 layers. The dose behind the laminated shield was calculated, and the potential application of this shielding under strict limitations on weight and dimensions was validated.

**Figure 6:**
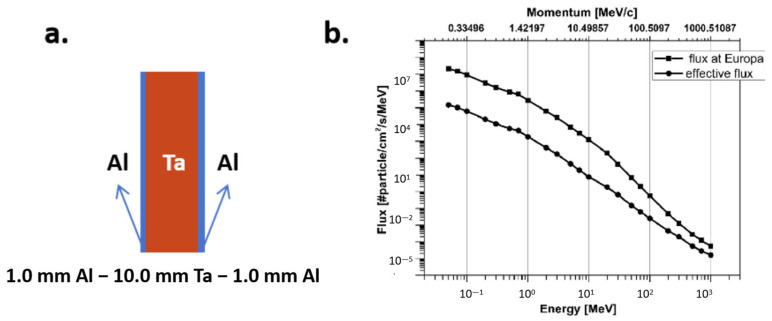
(**a**) Structure of the shielding material: 1.0mm Al–10.0mm Ta–1.0mm Al; (**b**) flux in the Europa environment and the predicted flux at the MCP active surface area with the Al-Ta-Al radiation shielding applied [99].

Tulej et al. [99] conducted a comprehensive study on the performance of a novel multi-layer shielding material (Al-Ta-Al) using both experimental and modeling approaches. The material, composed of rectangular plates (200 mm × 200 mm) arranged in an Al (1 mm)-Ta (10 mm)-Al (1 mm) sandwich structure or a circular (Ø 50 mm) Ta (10 mm) and Al (1 mm) configuration, was placed behind the Al (2 mm) entrance window of the vacuum chamber containing the MCP (microchannel plate) detector (Figure 6). Their research focused on its effectiveness as a radiation shield for incident electron beams in the momentum range of 17 to 345 MeV. Using beam diagnostic methods and an MCP detector designed for the NIM (Neutral Ion Mass spectrometer) instrument for the JUICE (the Jupiter Icy Moons Explorer) mission, they provided a quantitative analysis of the effective MCP detection efficiency for secondary particles and the values of the effective transmission coefficients in the applied incident electron energy range [99]. The Al-Ta-Al material demonstrated significant attenuation of high-energy electron beams. However, the interaction between the shielding material and the electron beam produced secondary radiation, primarily bremsstrahlung, which increased linearly with the incident electron energy and partially offset the initial attenuation effects. Notably, at electron energies around 130 MeV, the effective MCP particle rates of secondary radiation and incident electrons became equal, rendering the shielding ineffective. For higher energies, the shielding even amplified the incident beam. To address these challenges, they proposed introducing additional high-Z material layers to attenuate secondary radiation and optimizing the geometrical parameters of the MCP plate (channel pitch, length, and diameter) to reduce sensitivity to secondary radiation. These improvements were validated through experimental results, which confirmed the modeling predictions within the uncertainties of the investigated electron energy range.

Wang et al. [100] combined genetic algorithms with the MULASSIS (Multi-Layered Shielding Simulation Software) program to develop an optimization method for multi-layer material radiation protection, with the goal of increasing the absorbed dose after shielding. Using this method, the optimal shielding structure under certain weight constraints was mostly a combination of high- and low-atomic-number materials, typically in two or three layers, due to the dose enhancement phenomenon. Dose enhancement refers to the generation of bremsstrahlung radiation by high-energy electrons interacting with high-Z materials, which then backscatters at the interface between high-Z and low-Z materials, producing low-energy secondary electrons that contribute additional doses [100]. Generally, the more layers there are, the more pronounced the dose enhancement phenomenon becomes. Therefore, the optimal shielding structure should not have too many layers. During the design process, high-Z materials are placed on the outer side because their backscattering coefficient is greater than that of low-Z materials, allowing more high-energy particles to be reflected back into space. For 1 MeV electrons, the backscattering coefficient of Al is 12% under vertical incidence, while that of Ta is 48%. Thus, placing Ta material on the outer side is more advantageous for radiation protection.

Cheraghi et al. [101] initially fabricated PDMS (Polydimethylsiloxane)/Bi_2_O_3_ and PDMS/MWCNT (Multi-Walled Carbon Nanotube) nanocomposites with varying weight percentages (wt.%) of nanofillers. Flexible thin multi-layer nanocomposites with alternating layers of PDMS/Bi_2_O_3_ and PDMS/MWCNT were developed and characterized. By comparing the electron attenuation properties of PDMS/MWCNT, PDMS/Bi_2_O_3_, and PDMS/MWCNT/Bi_2_O_3_ nanocomposites with those of pure PDMS and Al, they investigated the effect of nanofillers embedded in the PDMS polymer matrix on shielding efficiency. The results showed that the addition of Bi_2_O_3_ and MWCNT significantly enhanced the electron attenuation capability of the pure polymer. Compared to Al, PDMS/MWCNT/Bi_2_O_3_ also demonstrated a weight advantage, as it could attenuate the same electron radiation energies with a lower areal density. However, to make it a promising candidate for electron radiation shielding in space applications, further improvements in thermal and mechanical properties were required. To achieve this, they optimized the nanocomposites by incorporating organized hexagonal boron nitride (OhBN) and the novel 2D nanomaterial MXene separately into the PDMS polymer matrix. A multi-layer nanocomposite structure was developed comprising PDMS/BN, PDMS/MXene, and PDMS/MWCNT/Bi_2_O_3_ layers, exhibiting enhanced thermal and shielding properties [101]. They fabricated this multi-layer structure with five different areal densities and evaluated its attenuation of high-energy electron radiation under electron beams with energies of 9, 12, 16, and 20 MeV. Given its high ratio of radiation shielding effectiveness to weight, this nanocomposite showed great potential as a space shielding material.

**Figure 7:**
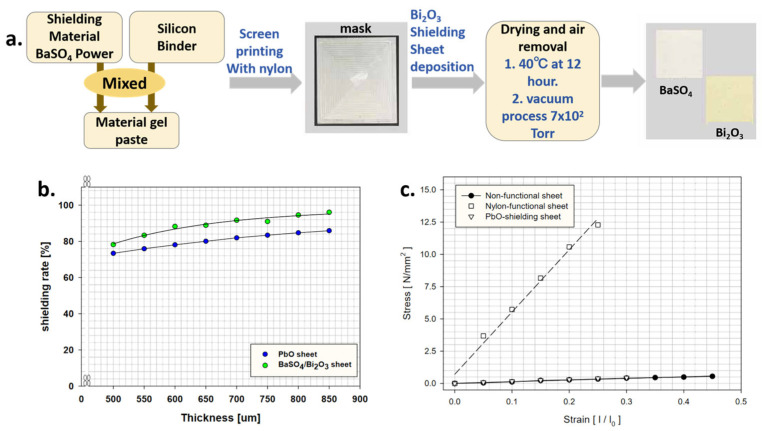
(**a**) Fabrication process of shielding sheet; (**b**) evaluation of shielding rate according to thickness change; (**c**) evaluation of tensile strength and elongation rate [102].

Heo et al. [102] developed a multi-layer structure by blending harmless materials like BaSO_4_, Bi_2_O_3_, and a Si binder, as shown in Figure 7. This approach ensured radiation shielding comparable to traditional Pb-based materials without the toxicity risks. Their study focused on enhancing physical properties while maintaining effective radiation protection. They tested a 40-degree shielding sheet with maximum porosity against conventional Pb sheets. The findings showed a shielding rate improvement of over 9% at equivalent thickness and demonstrated good mechanical properties. Additionally, the sheet containing BaSO_4_, nylon, and Bi_2_O_3_ demonstrated optimal physical characteristics.

**Figure 8:**
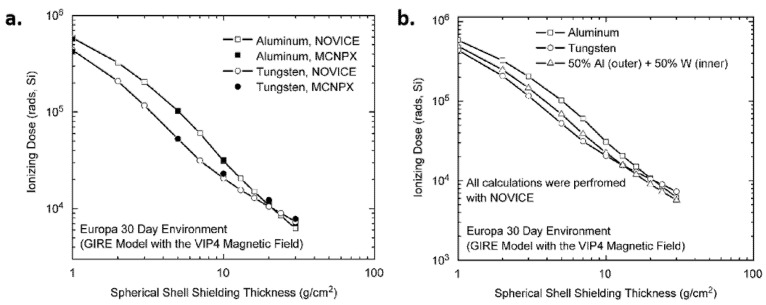
(**a**) Ionizing doses calculated for a detector located at the center of spherical shell shielding of Al and W on a 30-day Europa mission with NOVICE (a leading software suite for space systems radiation effects, https://empc.com/novice-software-2/, accessed on 29 May 2025) and Monte Carlo N Particle Transport Code eXtended (MCNPX), respectively; (**b**) ionizing doses calculated with NOVICE for a detector located at the center of the spherical shell shielding of Al, W, and 50% areal mass Al (outer layer)/50% areal mass W (inner layer) combination on a 30-day Europa mission (100 rad = 1 Gray) [104].

Spieth et al. [103] analyzed the radiation shielding effects of composite material electronic enclosures against high-energy space particles and found that composite enclosures with high-Z fillers can reduce weight by 30% compared to Al shielding and structures. Cherng et al. [104] showed that when the areal density was greater than 10 g/cm^2^, Al-W multi-layer shielding performed better than single-layer Al shielding (Figure 8).

➂ **Multi-layer materials in proton and electron shielding**

**Figure 9:**
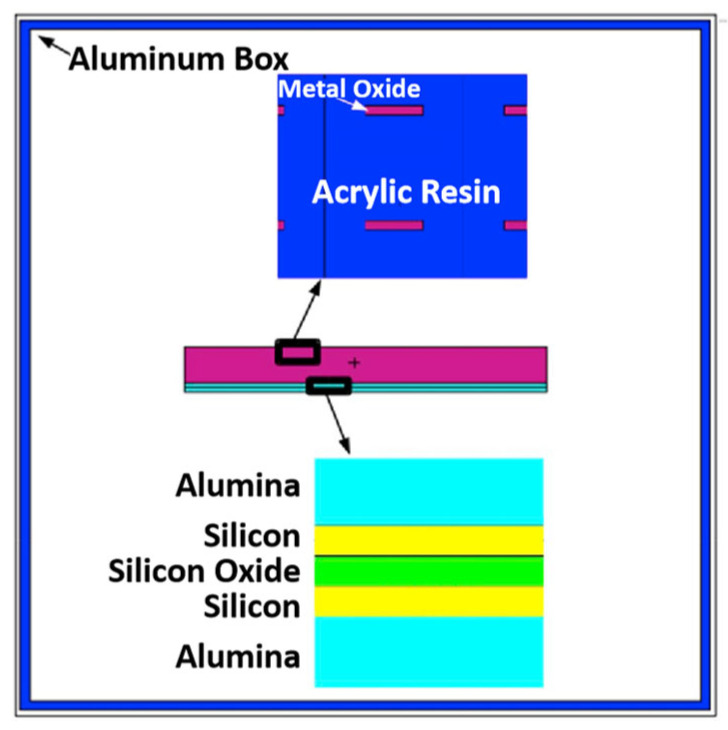
MCNP geometry input with 0.0508 cm (20 mils) Al enclosure [106].

The efficient shielding of protons and electrons can be achieved simultaneously by designing the materials, thickness, and order of layers in a multi-layer shielding system. For example, Vilkov et al. [105] prepared two types of multi-layer shielding materials with a total thickness of 1 mm using sodium silicate as the binder, W powder as the high-Z filler, and Al_2_O_3_ or BN as the low-Z filler. Radiation tests showed that W-Al_2_O_3_ and W-BN coatings significantly enhanced the attenuation of ionizing radiation flux and improved screening for proton electron irradiation. In addition, a research team from North Carolina State University [106] found that a radiation shielding material, as shown in Figure 9, made by adding rust to a protective coating can be lighter and more effective than Al.

Zhao et al. [107] used the MULYSSIS software package to calculate the shielding effects of four types of W-containing multi-layer structures on electrons and protons, as well as the total dose contributed by both, in Earth’s radiation environment. They pointed out that three-layer shielding materials perform better than two-layer structures. To address the issue that increased blocking leads to greater complexity in traditional packaging reinforcement, they proposed an electronic radiation shielding design for packaging radiation-hardened devices. By analyzing the GEO radiation environment and based on the interaction mechanism of high-energy electrons with shielding materials, they adopted an Al/Ta nanocomposite structure, combining high-/low-atomic-number materials [107]. The Al material was used to shield high-energy electrons, while the Ta material absorbed bremsstrahlung radiation, thereby shielding high-energy electron radiation and its secondary radiation in the space environment. The nano-Al/Ta composite coating prepared by cold spraying showed good shielding effects in practical tests, with a shielding efficiency of over 90% for composite coatings thicker than 0.4 mm and passed reliability tests.

**Figure 10:**
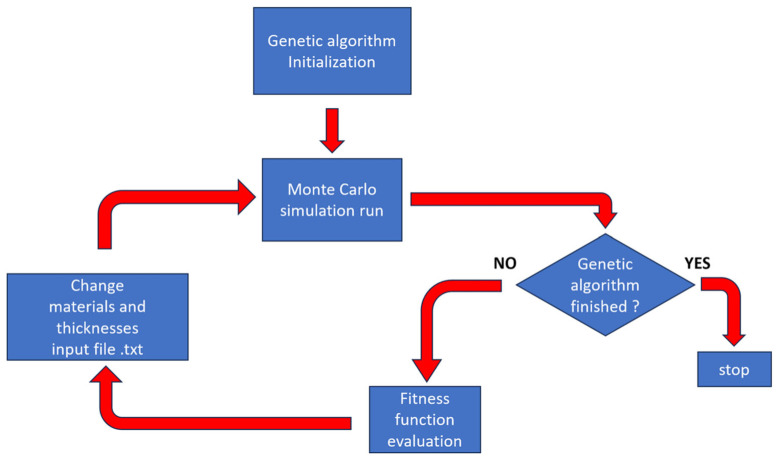
General flowchart of the genetic algorithm proposed with the embedded Monte Carlo simulation [108].

Recently, Cordella et al. [108] introduced a method based on the genetic algorithm, combined with the concept of the TID Database Vault and a customized parallel computing architecture, to optimize radiation shielding materials and their configurations, as shown in Table 3 and Figure 10.

The algorithm effectively minimized the TID on a Si slab while considering constraints such as the number of layers and the total thickness of the materials. After conducting over 43,000 simulations for impinging 14 MeV parallel neutrons, the best multi-layer shield configuration was identified under the constraints of a maximum thickness of 10.0 cm and a maximum of five layers. The optimal configuration was found to be as follows: (1, B_4_C, 6.0 cm), (2, Fe, 1.0 cm), (3, W, 1.0 cm), (4, Ta, 0.8 cm), and (5, W, 1.0 cm). This configuration can reduce the TID on the Si slab by nearly 38% compared to an air layer of the same thickness (9.8 cm).

Daneshvar et al. [97] conducted a study to design, optimize, and analyze various multi-layer shielding configurations for space environments dominated by electrons and protons. The research focused on identifying suitable metals to serve as effective local shields for protecting electronic components from radiation damage. The team used the MCNPX simulation method combined with the genetic algorithm to explore different material combinations and shielding designs. The study evaluated the performance of these multi-layer shields across various energy levels and space conditions, comparing them to a conventional 2 mm thick Al shielding layer. The results showed that the optimized multi-layer shields significantly outperformed traditional Al shields, achieving up to a 70% improvement in reducing the TID in electron environments and up to a 50% improvement in proton environments [109]. These advanced shields also effectively mitigated the impact of secondary radiations. The three-layer shield, in particular, emerged as a superior alternative to traditional Al shields due to its diverse material composition, reduced number of layers, and lower manufacturing costs. Under specific constraints, the optimal design was found to be a combination of bronze, Al, and Mo layers.

In Fetzer’s study [109], thousands of the multi-layer radiation shielding configurations were simulated against trapped particle spectra predicted for a geostationary transfer orbit. The goal was to demonstrate how material combinations and layer structures can be selected to minimize the TID inside nanosatellites with constrained mass budgets. The Geant4 Radiation Analysis for Space (GRAS) application was used to calculate ionizing dose deposition behind multi-layer shielding. Optimal multi-layer radiation shielding depends on various factors and must be tailored to specific radiation environments and mission requirements. The primary contributions are the methods presented for achieving this tailoring using open-source software and parallel computing. The multi-layer simulations performed for this work resulted in an extensive dataset for multi-layer shielding performance, enabling novel visualizations of the ionizing dose dependence on shielding composition based on quantitative results. The shielding simulations aimed to identify material combinations and layer structures which minimized the ionizing dose received inside small satellites on missions to Earth’s radiation belts. The Geant4 simulation toolkit was used with the GRAS application to compute the interaction of trapped particle spectra with various shielding materials arranged in multi-layer configurations with up to five layers [109].

The results showed that the TID received in Si plates behind multi-layer shielding was non-linearly dependent on the mass allocation between different materials in two- and three-layer shielding configurations. This indicates that optimizing the mass allocation in multi-layer shielding systems can lead to significantly lower TID than single-material shielding systems of the same total mass. Optimized polyethylene–Pb shields achieved up to 30% lower ionizing doses compared to an equal mass of either of the two materials or up to 50% lower compared to the same mass of Al. Especially for CubeSats, which have constrained mass budgets and a tendency to fail prematurely, this can mean mission extensions by several months or mass savings of several hundred grams, both of which are of great significance [109].

**Figure 11:**
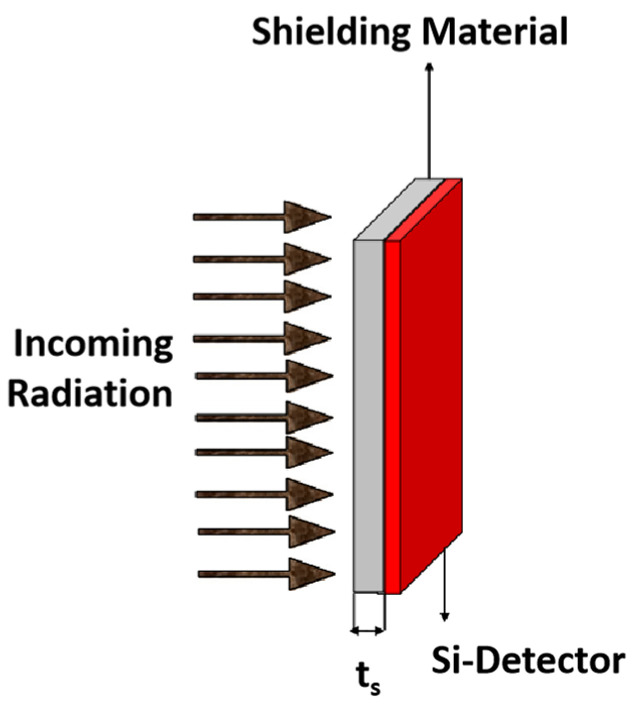
Schematic of shield geometry used in simulation (*t*_s_ = shield thickness) [110].

Emmanuel et al. [110] addressed the challenge of optimizing radiation shielding for spacecraft operating in three highly elliptical orbits (HEOs) considered for the PCW mission. They employed simulation methods to compare the shielding efficiency of five different materials (Figure 11), including high-Z materials (i.e., Ta and W) and low-Z polymer materials (e.g., polyethylene and epoxy). Their findings revealed that high-Z materials are more effective and lighter for shielding in electron-dominated environments, and low-Z polymers offer lighter and better shielding in proton-dominated environments. Specifically, polyethylene outperformed Al in electron-dominated environments, with a notable 22% weight reduction in proton-dominated environments. These weight savings, combined with enhanced spacecraft performance (e.g., better imaging, shorter communication delays) and extended mission duration (15 years), may justify the development of polymer-based shields to replace traditional Al shields.

**Figure 12:**
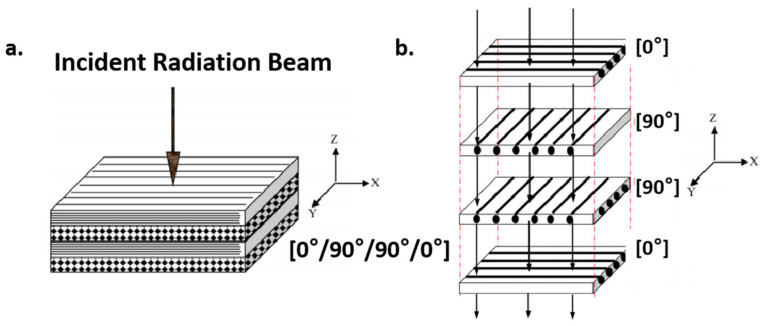
Schematic of (**a**) four-layer [0°/90°/90°/0°] composite shield and (**b**) attenuation paths for energetic particles transport through layers of the composite shield [111].

Emmanuel et al. [111] manufactured a layered polyethylene (PE) and graphite (G) composite shield to investigate its radiation shielding effectiveness and mechanical properties, as shown in Figure 12. The traditional Al shield, which attenuates radiation to a safe level for satellites, is heavier and scales with radiation intensity. In contrast, low-atomic-number materials like polyethylene can provide lighter shielding in high elliptical orbits compared to Al. However, polyethylene requires reinforcement with high-atomic-number fibers like graphite to enhance its mechanical strength. Through simulation, they explored the impact of graphite reinforcement on the mechanical properties and radiation shielding effectiveness of polyethylene. The TID deposited in a Si detector behind the composite shield was found to depend on the layer volume fraction, layer thickness, and stacking sequence of polyethylene and graphite layers. Configurations using polyethylene as the first layer to interact with incident radiation resulted in lower TID compared to those using graphite as the first layer. Non-symmetric stacking sequences also yielded lower TID than symmetric ones. The influence of layer thickness and volume fraction on TID was non-monotonic for both symmetric and non-symmetric configurations.

The study shows that one specific composite configuration ([PE/G]^2^ with 50% volume fraction of polyethylene and an areal density of 1 g/cm^2^) demonstrated a lower TID than pure polyethylene, showing that mechanical properties can be tailored with minimal negative impact on radiation shielding effectiveness. Although the composite structure was simplified to a layered model, the study highlights the significant influence of structural design on the overall radiation shielding performance of polyethylene-based composites [111].

Gohel et al. [112] employed a multi-layered shielding approach to mitigate radiation exposure from trapped radiation, Solar Particle Events (SPEs), and Galactic Cosmic Radiation (GCR). They utilized a variety of shielding materials, including Al, polyethylene, H-storing boron nitride, water, polybenzoxazine, epoxy, Kevlar, Zylon, polyetherimide, polysulfone, lithium hydride (LiH), liquid methane, and Hated graphite nanofibers (HGNFs). Using the On-line Tool for the Assessment of Radiation in Space (OLTARIS) and the High Charge and Energy TRaNsport (HZETRN) model, they calculated the dose equivalent (DE) for these materials under the 2010 solar minimum GCR conditions. Additionally, they analyzed the dose equivalent for combinations of a 10 g/cm^2^ Al slab [112]. Their analysis revealed that the combination of Al and lithium hydride (Al + LiH) was particularly effective in reducing the dose equivalent compared to other materials. They also explored combinations of H-, B-, and N-rich material slabs placed behind an Al slab. The results showed that the first H-rich material layer in the shield provided a higher dose equivalent reduction per unit thickness than any other material layer. In sandwich configurations, materials like PE, LiH, liquid methane, and H-storing BN were placed between Al layers, with Al + LiH + Al demonstrating superior performance. Comparisons were also made between the dose equivalents calculated using OLTARIS and HZETRN transport codes. Through multi-layered and complex trial analyses, they found that combinations like Al + LiH and Al + liquid methane significantly reduced dose equivalents. In sandwich configurations, Al + LiH + Al outperformed the previously proposed Al + PE + Al. In complex multi-layered trials, GCR and secondary neutron radiation were absorbed by polyethylene and H-storing B nitride after interacting with Al. At a shielding thickness of 45 g/cm^2^, the dose equivalent was reduced to 0.41 mSv/year for the Al + LiH combination, while complex trial configurations 3 (APPPPBBLL) and 6 (ABBBBPPLL) achieved a dose equivalent of 0.39 mSv/year. Their findings suggested that using diverse material combinations is more effective in reducing dose equivalents in lower-thickness regions compared to combinations of just two materials [112].

**Figure 13:**
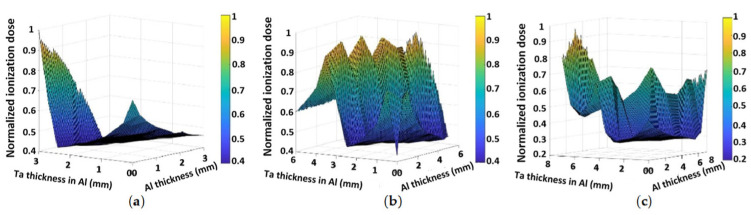
Ionization dose of various particles predicted by the FY-3 satellite orbit after multi-layer shielding. At a certain total thickness, TID was obtained by different thickness ratios of three materials. The X and Y coordinates represent the equivalent Al thickness of Ta and Al thickness, respectively, while the remaining equivalent Al thickness is assigned to HDPE. To facilitate comparison and comprehension, the maximum TID is used as a normalization benchmark. It is evident from the figure that TID is significantly different for the various proportions of Al, Ta, and HDPE under the same weight at (**a**) a total thickness of 3 mm; (**b**) a total thickness of 5 mm; and (**c**) a total thickness of 7 mm [112].

To optimize the protection strategy, Han et al. [113] employed the Monte Carlo method to calculate the energy loss of electrons and protons in materials such as Al, Ta, and high-density polyethylene (HDPE) (Figure 13). They found when the equivalent Al thicknesses of Al, Ta, and HDPE are 0.7 mm, 2.8 mm, and 3.5 mm, respectively, the total equivalent Al thickness is 7 mm, yielding the lowest TID in the object, which is 71% lower than the maximum dose. However, for total equivalent Al thicknesses of 3 mm or 5 mm, even if the three materials reach the best mass ratio, the lowest ionization dose in the object is only 57% lower than the highest dose. Through this analysis, the optimal mass ratio of Al, Ta, and HDPE was determined to achieve the lowest ionizing dose in the particle environment of the FY-3 satellite orbit. This protection strategy provides valuable design concepts for high-sensitivity photodetection instruments.

Multi-layer shielding materials have also been applied and tested on the International Space Station. Research has shown that multi-layer structures composed of aluminum and polyethylene offer better shielding performance than single-layer aluminum materials of the same thickness. This type of multi-layer structure can effectively reduce electron penetration and enhance overall shielding efficiency [114].

The concept of multi-layer structures is indeed promising for various applications. However, potential issues such as delamination and thermal mismatch between layers are crucial for their successful implementation. In space environments, both extreme temperature variations and mechanical stresses are common. These issues can lead to reduced structural integrity and performance [115]. For example, the thermal mismatch due to different coefficients of thermal expansion in each layer can cause significant thermal stress, leading to buckling, delamination, and other failures [116]. To mitigate these issues, the careful selection of material interfaces is essential. Some studies suggest that interfaces with engineered heterophase boundaries (e.g., between immiscible phases) can be more stable and effective in trapping and annihilating radiation-induced defects [117]. For instance, Cu/Ni multi-layer structures have been investigated for their deformation behavior and stability under irradiation, showing that certain interface structures can enhance radiation tolerance [118].

Radiation-induced point defects, such as interstitials and vacancies, can significantly impact the performance of multi-layer structures. These defects can aggregate to form voids or dislocation loops, leading to material hardening and embrittlement [117]. However, interfaces can act as sinks for these defects, reducing their clustering and improving radiation tolerance. For example, Cu/Ag nanocomposites have been studied for their radiation behavior, demonstrating that interfaces can heal radiation defects in certain layers [119].

In summary, passive shielding works best against the lowest-energy particles that do not produce secondaries. And it can be concluded that multi-layer materials, due to their structural characteristics, are able to more effectively block and attenuate the irradiation of high-energy charged particles, thereby achieving superior shielding effects. While multi-layer structures offer advantages in various applications, addressing delamination and thermal mismatch is critical. Further research and development in this area are necessary to optimize material interfaces and improve the overall reliability of multi-layer structures for space applications.

## 4. Future Research Prospects

### 4.1. Advantages

Passive shielding material offer several advantages, including simple structure and high reliability. They rely on the shielding properties of physical materials without the need for complex electronic devices or control systems, making them highly effective in long-term operations. Additionally, since they do not depend on external energy sources, they have low operational and maintenance costs, further reducing the economic burden of space missions.

### 4.2. Disadvantages

The materials are often heavy, significantly increasing the weight of spacecraft and thereby affecting their payload capacity. Moreover, passive shielding has limited effectiveness against high-energy particles, making it difficult to completely block high-energy protons and electrons from cosmic rays. Despite these limitations, passive shielding materials remain valuable in specific orbital environments. For example, in both LEO and MEO environments, where particle energies are relatively low, passive shielding materials can provide effective protection without imposing significant weight or cost burdens on spacecraft. In the GEO environment, combining multi-layer shielding structures can enhance protection and optimize shielding performance. Even in the more complex radiation environment of the geostationary transfer orbit, challenges posed by high-energy particles can be addressed through optimized material and structural designs, ensuring the safe operation of spacecraft [120].

### 4.3. Future Research Directions

With the continuous advancement of technology, the development of lighter and more efficient new shielding materials has become a key focus of current research. In complex radiation environments, the radiation resistance and mechanical strength of materials are of utmost importance. In recent years, researchers have developed shielding materials with higher mechanical properties and radiation resistance by combining metals, polymers, C-based materials, and other conductive additives [121]. These materials, by combining high-Z and low-Z elements, achieve efficient shielding of protons and electrons while balancing mechanical strength and shielding performance.

In addition, optimizing the design of multi-layer shielding structures is key to enhancing shielding effectiveness. By combining numerical simulations and experimental studies, the design of multi-layer shielding structures can be optimized to perform well in different orbital environments [122].

Furthermore, the future development of passive shielding materials should increasingly focus on deep integration with other protective technologies. On the one hand, the deep integration of passive shielding technology with spacecraft design can enhance overall protective performance. By optimizing the distribution and structural design of materials, the radiation resistance of spacecraft can be significantly improved without adding extra weight. On the other hand, future research directions should include the application of new materials, structural optimization, and the introduction of intelligent testing technologies. For example, the use of 3D printing and micro-/nano-processing technologies can improve the controllability and flexibility of the manufacturing process [123,124,125].

## 5. Conclusions

Passive shielding materials are crucial for protecting spacecraft from high-energy charged particles. They have good performance in both LEO and MEO environments with lower particle energies but face challenges in high-energy environments like GEO, where high-energy protons and electrons have strong penetration capabilities. Therefore, further optimization of materials and structural designs is needed.

The development of passive shielding materials should focus on several key areas. First, research into new shielding materials, such as nanocomposites, high-performance polymers, and new metal alloys, should be essential for improving protection without increasing the spacecraft’s weight. Second, optimizing multi-layer shielding structures through numerical simulations and experimental validation can provide customized solutions for different orbital environments.

Integrating passive shielding materials into the initial design phase of spacecraft can enhance overall protection performance. This includes optimizing the distribution and combination of shielding materials. Additionally, combining passive shielding with other protective technologies, like radiation-hardened electronics, can provide more reliable protection in complex environments.

In summary, passive shielding materials are vital for spacecraft protection. However, with the increasing complexity of space missions, continuous innovation and optimization are required. By exploring new materials, optimizing structural designs, and strengthening synergistic applications, passive shielding materials can provide a solid foundation for the safe operation of future spacecraft.

## Figures and Tables

**Table 1:** Average parameters of particles fluxes of cosmic rays and Earth’s radiation belts [27].

Type of Corpuscular Radiation	Composition	Energy of Particles, MeV	Flux Density, m^−2^s^−1^
Galactic cosmic ray	Protons, helium nuclei, and heavier nuclei	10^2^ to 10^14^	1.5 × 10^4^
		(For all groups of nuclei)	1 × 10^3^
			1.2 × 10^1^
Solar cosmic rays	Protons	1 to 10^4^	10^7^ to 10^8^
Earth’s radiation belts	Protons	1 to 30	3 × 10^11^
		>30	2 × 10^8^
	Electrons	0.1 to 1.0	1 × 10^12^
		>1.0	1 × 10^10^

**Table 2:** Atomic number, density, mass efficiency, shielding efficiency, cost, manufacturability, and mechanical properties of Al, polyethylene, Pb, Fe, W, and Ta.

Material	Atomic Number	Density (g/cm^3^)	Mass Efficiency	Shielding Efficiency	Cost	Manufacturability	Mechanical Properties
**Al**	13	2.7	High mass efficiency due to low density	Provides some shielding against high-energy electrons, but poor shielding against high-energy protons	Low cost	Excellent manufacturability, easy to process and form	Moderate strength and hardness, with good ductility and toughness
**Polyethylene**	-	0.91 to 0.96	High mass efficiency due to low density	Limited shielding against high-energy protons and electron	Low cost	Excellent manufacturability, can be processed using various methods	Mechanical properties vary with density: low-density polyethylene is flexible, while high-density polyethylene has higher strength and hardness
**Pb**	82	11.34	Low mass efficiency due to high density	Good shielding against high-energy electrons, but limited shielding against high-energy protons	Relatively high cost	Average manufacturability, requires special handling during processing	Soft and ductile, but with low strength
**Fe**	26	7.8	Moderate mass efficiency	Provides some shielding against high-energy electrons, but limited shielding against high-energy protons	Low cost	Excellent manufacturability, easy to process and form	High strength and hardness, with good toughness
**W**	74	19.35	Low mass efficiency due to high density	Good shielding against both high-energy electrons and protons, but requires thicker material to achieve ideal shielding	High cost	Poor manufacturability, difficult to process	Very high strength and hardness, but also brittle
**Ta**	73	22.59	Low mass efficiency due to high density	Good shielding against both high-energy electrons and protons, but requires thicker material to achieve ideal shielding	High cost	Poor manufacturability, difficult to process	High strength and hardness, with good toughness

**Table 3:** Material configuration list used for the multi-layer shielding analysis [108].

Material	Min Thickness (cm)	Max Thickness (cm)	Step (cm)	Density [g/cm^3^]
B_4_C	2.0	10.0	2.0	2.52
LiF	2.0	10.0	2.0	2.64
Paraffin	2.0	10.0	2.0	0.93
Polyethylene	2.0	10.0	2.0	0.94
Borotron	2.0	10.0	2.0	1.00
Al	2.0	10.0	2.0	2.70
Ta	0.2	1.0	0.2	16.65
W	0.2	1.0	0.2	19.30
Pb	0.2	1.0	0.2	11.35
Cu	0.2	1.0	0.2	8.96
Fe	0.2	1.0	0.2	7.87

## Data Availability

No new data were created or analyzed in this study.

## References

[1] Baker C.J., Simske S.J. (2024). Extension to critical analysis of active shielding methods for space radiation protection. Innovation in MIMO Technologies, Systems, and Antennas.

[2] Yang X.N. (2024). Challenges and opportunities of spacecraft environment engineering in the new era. Spacecr. Environ. Eng..

[3] Zeynali O., Masti D., Gandomkar S. (2012). Shielding protection of electronic circuits against radiation effects of space high energy particles. Adv. Appl. Sci. Res..

[4] Kang N., Zhang L., Zong W., Huang P., Zhang Y., Zhou C., Qiao J., Xue B. (2024). A multi-satellite space environment risk Prediction and real-time warning system for satellite safety management. Remote Sens..

[5] Berger T., Burmeister S., Matthiä D., Przybyla B., Reitz G., Bilski P., Hajek M., Sihver L., Szabo J., Ambrozova I. (2017). 3D: Radiation measurements with the DOSTEL instruments onboard the Columbus Laboratory of the ISS in the years 2009–2016. J. Space Weather Space Clim..

[6] Bahadori A.A. (2024). Space radiation protection in the modern era: New approaches to familiar challenges. Radiat. Phys. Chem..

[7] Zeitlin C., Castro A.J., Beard K.B., Abdelmelek M., Hayes B.M., Johnson A.S., Stoffle N., Rios R.R. (2023). Results from the radiation assessment detector on the international space station: Part 1, the charged particle detector. Life Sci. Space Res..

[8] George S.P., Gaza R., Matthiä D., Laramore D., Lehti J., Campbell-Ricketts T., Kroupa M., Stoffle N., Marsalek K., Przybyla B. (2024). Space radiation measurements during the Artemis I lunar mission. Nature.

[9] Mohanty P.K. (2025). Cosmic ray sources and detectors. Eur. Phys. J. Spec. Top..

[10] Zweibel E.G. (2017). The basis for cosmic ray feedback: Written on the wind. Phys. Plasmas.

[11] Liu L., Dong Z., Su H., Yu D. (2021). A study of distributed earth observation satellites mission scheduling method based on game-negotiation mechanism. Sensors.

[12] Liu X., Laporte G., Chen Y., He R. (2017). An adaptive large neighborhood search metaheuristic for agile satellite scheduling with time-dependent transition time. Comput. Oper. Res..

[13] Gadisa D., Tafes E. (2025). Design and on-orbit performance evaluation of Ethiopian earth observation satellite multispectral optical imaging payload. Opt. Laser Technol..

[14] Krigman S., Grinshpoun T., Dery L. (2024). Scheduling of Earth observing satellites using distributed constraint optimization. J. Sched..

[15] Thomsen D.L., Cano R.J., Jensen B.J., Hales S.J., Alexa J.A. (2018). Methods of Making Z-Shielding. U.S. Patent.

[16] Vishwakarma S., Chauhan A.S., Aasma S. (2014). A comparative study of satellite orbits as low Earth orbit (LEO) and geostationary Earth orbit (GEO). J. Phys. Sci. Eng. Technol..

[17] Selva D., Krejci D. (2012). A survey and assessment of the capabilities of Cubesats for Earth observation. Acta Astronaut..

[18] Sweeting M.N. (2018). Modern small satellites-changing the economics of space. Proc. IEEE.

[19] Ginet G.P., O’Brien T.P., Huston S.L., Johnston W.R., Guild T.B., Friedel R., Lindstrom C.D., Roth C.J., Whelan P., Quinn R.A. (2014). AE9, AP9 and SPM: New models for specifying the trapped energetic particle and space plasma environment. Space Sci. Rev..

[20] Perren B.B., Kaiser J., Arz H.W., Dellwig O., Hodgson D.A., Lamy F. (2025). Poleward displacement of the Southern Hemisphere Westerlies in response to Early Holocene warming. Commun. Earth Environ..

[21] Narici L., Berger T., Matthiä D., Reitz G. (2015). Radiation measurements performed with active detectors relevant for human space exploration. Front. Oncol..

[22] Johnson N.L. Medium Earth Orbits: Is there a need for a third protected region. Proceedings of the 61st International Astronautical Congress.

[23] Zhang B., Zhang S., Shen G., Tuo C., Zhang X., Zhang H., Quan L., Tian C., Hou D., Zhou P. (2023). Monitor of the single event upsets and linear energy transfer of space radiation on the Beidou navigation satellites. Open Astron.

[24] He W.P., Wei Q.F. (2006). Space environmental factors influencing on GEO satellite’s life and reliability and their evaluation, verification and guarantee technologies. Spacecr. Environ. Eng..

[25] Dodd P.E., Massengill L.W. (2003). Basic mechanisms and modeling of single-event upset in digital microelectronics. IEEE Trans. Nucl. Sci..

[26] Baumann R.C. (2005). Radiation-induced soft errors in advanced semiconductor technologies. IEEE Trans. Device Mater. Reliab..

[27] Samwel S.W., El-Aziz E.A., Garrett H.B., Hady A.A., Ibrahim M., Amin M.Y. (2019). Space radiation impact on smallsats during maximum and minimum solar activity. Adv. Space Res..

[28] Dunai T.J. (2010). Cosmogenic Nuclides: Principles, Concepts and Applications in the Earth Surface Sciences.

[29] Ingo L., Hirtz J., David J.C. (2021). Galactic cosmic rays, cosmic-ray variations, and cosmogenic nuclides in meteorites. Astrophys. J..

[30] Peron G., Casanova S., Gabici S., Baghmanya V., Aharonian F. (2024). The contribution of winds from star clusters to the Galactic cosmic-ray population. Nat. Astron..

[31] Tatischeff V., Raymond C., Duprat J., Gabic S., Recchia S. (2021). The origin of Galactic cosmic rays as revealed by their composition. Mon. Not. R. Astron. Soc..

[32] Fu S., Zhang X., Zhao L., Li Y. (2021). Variations of the galactic cosmic rays in the recent solar cycles. Astrophys. J. Suppl. Ser..

[33] An Q., Asfandiyarov R., Azzarello P., Bernardini P., Bi X.J., Cai M.S., Chang J., Chen D.Y., Chen H.F., Chen J.L. (2019). Measurement of the cosmic ray proton spectrum from 40 GeV to 100 TeV with the DAMPE satellite. Sci. Adv..

[34] Varsi F., Ahmad S., Chakraborty M., Chandra A., Dugad S.R., Goswami U.D., Gupta S.K., Hariharan B., Hayashi Y., Jagadeesan P. Cosmic ray energy spectrum and composition measurements from the GRAPES-3 experiment: Latest results. Proceedings of the 37th International Cosmic Ray Conference (ICRC 2021).

[35] Cannady N., Asaoka Y., Satoh F., Tanaka M., Torii S., Cherry M.L., Mori M., Adriani O., Akaike Y., Asano K. (2018). Characteristics and performance of the CALorimetric Electron Telescope (CALET) calorimeter for gamma-ray observations. Astrophys. J. Suppl. Ser..

[36] Aguilar M., Ali Cavasonza L., Ambrosi G., Arruda L., Attig N., Barao F., Barrin L., Bartoloni A., Başeğmez-du Pree S., Bates J. (2021). The Alpha Magnetic Spectrometer (AMS) on the international space station: Part II—Results from the first seven years. Phys. Rep..

[37] Durante M., Cucinotta F.A. (2011). Physical basis of radiation protection in space travel. Rev. Mod. Phys..

[38] Zeitlin C., Hassler D.M., Cucinotta F.A., Ehresmann B., Wimmer-Schweingruber R.F., Brinza D.E., Kang S., Weigle G., Böttcher S., Böhm E. (2013). Measurements of energetic particle radiation in transit to Mars on the Mars Science Laboratory. Science.

[39] Schwadron N.A., Blake J.B., Case A.W., Joyce C.J., Kasper J., Mazur J., Petro N., Quinn M., Porter J.A., Smith C.W. (2014). Does the worsening galactic cosmic radiation environment observed by CRaTER preclude future manned deep space exploration. Space Weather.

[40] Desai M., Giacalone J. (2016). Large gradual solar energetic particle events. Living Rev. Sol. Phys..

[41] Ahmad N., Herdiwijaya D., Djamaluddin T., Usui H., Miyake Y. (2018). Diagnosing low earth orbit satellite anomalies using NOAA-15 electron data associated with geomagnetic perturbations. Living Rev. Sol. Phys..

[42] Miś T.A., Pytlak D., Kościanek B., Szalkowski K., Czerniej J., Kucharczyk P., Salamon M., Płosko M., Styrna K., Wąsowska S. (2025). Electrical response of photovoltaic power cells to cosmic radiation in the stratosphere. Electronics.

[43] Stepanova M., Pinto V., Antonova E. (2024). Regarding the relativistic electron dynamics in the outer radiation belt: A historical view. Rev. Mod. Plasma Phys..

[44] Thorne R.M., Li W., Ni B., Ma Q., Bortnik J., Chen L., Baker D.N., Spence H.E., Reeves G.D., Henderson M.G. (2013). Rapid local acceleration of relativistic radiation-belt electrons by magnetospheric chorus. Nature.

[45] Li W., Thorne R.M., Ma Q., Ni B., Bortnik J., Baker D.N., Spence H.E., Reeves G.D., Kanekal S.G., Green J.C. (2014). Radiation belt electron acceleration by chorus waves during the 17 March 2013 storm. J. Geophys. Res..

[46] Baker D.N., Erickson P.J., Fennell J.F., Foster J.C., Jaynes A.N., Verronen P.T. (2018). Space weather effects in the Earth’s radiation belts. Space Sci. Rev..

[47] Turner D.L., Kilpua E.K.J., Hietala H., Claudepierre S.G., O’Brien T.P., Fennell J.F., Blake J.B., Jaynes A.N., Kanekal S., Baker D.N. (2019). The response of Earth’s electron radiation belts to geomagnetic storms: Statistics from the Van Allen Probes era including effects from different storm drivers. J. Geophys. Res..

[48] Guo J., Zeitlin C., Wimmer-Schweingruber R.F., Hassler D.M., Ehresmann B., Rafkin S., Forstner von Freiherr J.L., Khaksarighiri S., Liu W., Wang Y. (2021). Radiation environment for future human exploration on the surface of Mars: The current understanding based on MSL/RAD dose measurements. Astron. Astrophys. Rev..

[49] Nwankwo V.U.J., Jibiri N.N., Kio M.T. (2020). The impact of space radiation environment on satellites operation in near-Earth space. Satellites Missions and Technologies for Geosciences.

[50] Buitrago-Leiva J.N., El Khayati Ramouz M., Camps A., Ruiz-de-Azua J.A. (2024). Statistical analysis of LEO and GEO satellite anomalies and space radiation. Aerospace.

[51] Veldhuis S.A., Boix P.P., Yantara N., Li M., Sum T.C., Mathews N., Mhaisalkar S.G. (2016). Perovskite materials for light-emitting diodes and lasers. Adv. Mater..

[52] Seo E.S., Anderson T., Angelaszek D., Baek S.J., Baylon J., Buénerd M., Copley M., Coutu S., Derome L., Fields B. (2014). Cosmic ray energetics and mass for the international space station (ISS-CREAM). Adv. Space Res..

[53] Tan Z.K., Moghaddam R.S., Lai MLDocampo P., Higler R., Deschler F., Price M.i., Sadhanala A., Pazos L.M., Credgington D., Hanusch F. (2014). Bright light-emitting diodes based on organometal halide perovskite. Nat. Nanotechnol..

[54] Schwank J.R., Shaneyfelt M.R., Fleetwood D.M., Felix J.A., Dodd P.E., Paillet P., Ferlet-Cavrois V. (2008). Radiation effects in MOS oxides. IEEE Trans. Nucl. Sci..

[55] Was G.S. (2007). Fundamentals of Radiation Materials Science: Metals and Alloys.

[56] Zheng M., Adolphi F., Ferrachat S., Mekhaldi F., Lu Z., Nilsson A., Lohmann U. (2024). Modeling atmospheric transport of cosmogenic radionuclide 10Be using geos-chem 14.1. 1 and echam6. 3-ham2. 3: Implications for solar and geomagnetic reconstructions. Geophys. Res. Lett..

[57] Schwenn R. (2006). Space weather: The solar perspective. Living Rev. Sol. Phys..

[58] Spillantini P. (2010). Active shielding for long duration interplanetary manned missions. Adv. Space Res..

[59] Akçalı Ö., Toker O., Bilmez B., Bilmez B., İçelli O. (2021). Efficiency analysis of multiple detector effects on MCNP 6.2 simulations. Prog. Nucl. Energy.

[60] Song T.H., Wei X.C., Ju J.J., Liang W.T., Gao R.X. (2022). An effective EMI source reconstruction method based on phaseless near-field and dynamic differential evolution. IEEE Trans. Electromagn. Compat..

[61] Lecoq P. (2020). Scintillation detectors for charged particles and photons. Particle Physics Reference Library: Volume 2: Detectors for Particles and Radiation.

[62] Novikov L.S., Mileev V.N., Voronina E.N., Galanina L.I., Makletsov A.A., Sinolits V.V. (2009). Radiation effects on spacecraft materials. J. Surf. Investig. X-Ray Synchrotron Neutron Tech..

[63] Sajida M., Checheninb N.G., Torresc F.S., Hanifd M.N., Gulzarie U.A., Arslanf S., Khang E.U. (2018). Analysis of total ionizing dose effects for highly scaled CMOS devices in low earth orbit. Nucl. Instrum. Methods Phys. Res. B.

[64] Vahedi Z., Ezzati A.O., Sabri H. (2024). Design of a space radiation shield for electronic components of LEO satellites regarding displacement damage. Eur. Phys. J. Plus.

[65] Borts B.V., Bratchenko M.I., Dyuldya S.V., Marchenko I.G., Sanzharevsky D.A., Tkachenko V.I. (2014). Monte carlo evaluation of the radiation shielding efficiency of laminated composites under electron and photon irradiation. Eur. Phys. J. E.

[66] Yuan S., Zhang S., Wei J., Gao Y., Zhu Y., Wang H. (2024). Materials selection, design, and regulation of polymer-based H barrier composite coatings, membranes and films for effective H storage and transportation: A comprehensive review. Int. J. Hydrog. Energy.

[67] Sobhiga G., Maria H.J., Mozeti M., Thomas S. (2024). A review on green materials: Exploring the potential of poly (vinyl alcohol) (PVA) and nanocellulose composites. Int. J. Biol. Macromol..

[68] Thibeault S.A., Fay C.C., Lowther S.E., Earle K.D., Sauti G., Kang J.H., Park C., McMullen A.M. (2012). Radiation Shielding Materials Containing Hydrogen, Boron, and Nitrogen: Systematic Computational and Experimental Study.

[69] Zhang K., Zhu B., Du C.B., Han Y. (2024). Preparation and radiation shielding mechanism of lead-boron-polyethylene composites. Chin. J. Appl. Chem..

[70] Fujita Y., Myojoyama A., Saitoh H. (2015). Bremsstrahlung and photoneutron production in a steel shield for 15-22-MeV clinical electron beams. Radiat. Prot. Dosim..

[71] Fujimoto T., Monzen H., Nakata M., Okada T., Yano S., Takakura T., Kuwahara J., Sasaki M., Higashimura K., Hiraoka M. (2014). Dosimetric shield evaluation with W sheet in 4, 6, and 9áMeV electron beams. Phys. Med..

[72] Lasi D., Tulej M., Meyer S., Lüthi M., Galli A., Piazza D., Wurz P., Reggiani D., Xiao H., Marcinkowski R. (2016). Shielding an MCP detector for a space-borne mass spectrometer against the harsh radiation environment in Jupiter’s magnetosphere. IEEE Trans. Nucl. Sci..

[73] Zhao K., Wen C., Sang J., Bai J.Y., Liu F., Sun H.R., Lin P.T., Zhang L.G. (2021). Irradiation passive protection technology for space electronic products. Space Electron. Technol..

[74] El-Samrah M.G., Nabil I.M., Shamekh M.E., Elmasry M., Osman M. (2024). Microstructure and radiation shielding capabilities of Al-Cu and Al-Mn alloys. Sci. Rep..

[75] Nambiar S., Yeow J.T.W. (2012). Polymer-composite materials for radiation protection. ACS. Appl. Mater. Interfaces.

[76] Abd El-Hameed A.M. (2022). Radiation effects on composite materials used in space systems: A review. J. Astron. Geophys..

[77] Ahmed B., Shah G.B., Malik A.H., Aurangzeb, Rizwan M. (2020). Gamma-ray shielding characteristics of flexible Sie W composites. Appl. Radiat. Isot..

[78] Srilakshmi B., Reddy A.J., Ambika M., Kuttukaran S.S., Reddy Y.H., Nagaiah N., Deepika D., Prashantha K. (2025). Exploration of the gamma-ray shielding capabilities of W oxide and chromium-Infused sie rubber composites. Radiat. Phys. Chem..

[79] Brock A., Mooney-Rivkin M., Idziak L., Salas A., Wrbanek S., Wrbanek J. Development of a universal small-Satellite payload for on-orbit characterization and evaluation of novel radiation-shielding materials. Proceedings of the AIAA SCITECH 2025 Forum.

[80] Bachir G., Abdechafik H., Mecheri K. Comparison electromagnetic shielding effectiveness between single layer and multilayer shields. Proceedings of the 2016 51st International Universities Power Engineering Conference (UPEC).

[81] Jose S.A., Cowan N., Davidson M., Godina G., Smith I., Xin J., Menezes P.L. (2025). A comprehensive review on cellulose Nanofibers, nanomaterials, and composites: Manufacturing, properties, and applications. Nanomaterials.

[82] Jose S.A., Cowan N., Davidson M., Godina G., Smith I., Xin J., Menezes P.L. (2016). Electromagnetic interference shielding with 2D transition metal carbides (MXenes). Science.

[83] Srivastava S.K., Manna K. (2022). Recent advancements in the electromagnetic interference shielding performance of nanostructured materials and their nanocomposites: A review. J. Mater. Chem. A Mater..

[84] Nan Z., Wei W., Lin Z.H., Ouyang J.Y., Chang J.J., Hao Y. (2024). Flexible electromagnetic interference shields: Materials, structure and multifunctionalization. Mater. Sci. Eng. R Rep..

[85] Mansoori M., Luo S., Choi D. (2025). Fabrication and characterization of the flexible three-layered thin film composites based on MXene/Fe_3_O_4_/MWCNT for electromagnetic shielding applications. Compos. Part C Open Access.

[86] Kaushik N., Singh P., Rana S., Sahoo N.G., Ahmad F., Jamil M. (2024). Self-healable electromagnetic wave absorbing/shielding materials for stealth technology: Current trends and New frontiers. Mater. Today Sustain..

[87] Zhang X.L., Deng Z.S., Song H.Z., Guo M.H., Li L. (2024). Liquid metal electromagnetic wave shielding and absorbing film for solving electromagnetic interference in flexible sensors. Sci. China Mater..

[88] Rao K.S.S., Reddy C.J., Krishna R.S.S.S.S.G.N., Subhakar V.R.S., Vignesh R.V., Govindaraju M. (2025). Mechanical and corrosion characteristics of low-density polyethylene reinforced with Al alloy AA6065 honeycomb structure. Polym. Bull..

[89] Al-Saleh W.M., Almutairi H.M., Sayyed M.I., Elsafi M. (2023). Multilayer radiation shielding system with advanced composites containing heavy metal oxide nanoparticles: A free-lead solution. Sci. Rep..

[90] Yang F., Li Z., Liu Z., Zhuang Z. (2021). Shock loading mitigation performance and mechanism of the PE/wood/PU/foam structures. Int. J. Impact Eng..

[91] Al Zaman M.A., Nizam Q.M.R. (2022). Study on shielding effectiveness of a combined radiation shield for manned long termed interplanetary expeditions. J. Space Saf. Eng..

[92] DeWitt J.M., Benton E.R. (2024). Secondary proton buildup in space radiation shielding. Life Sci. Space Res..

[93] Almuqrin A.H., Sayyed M.I., Alorain D.A., Elsaf M. (2024). Multilayer radiation shields for nuclear and radiological centers using free-lead materials and nanoparticles. Ann. Nucl. Energy.

[94] Tong X.C. (2016). Advanced Materials and Design for Electromagnetic Interference Shielding.

[95] Hu S., Han P., Meng C., Yu Y., Han S., Wang H., Wei G., Gu Z. (2024). Flexible and ultrathin MXene/nanofiber composite films by self-stacking assembly for enhanced electromagnetic interference shielding performance. Appl. Surf. Sci..

[96] Singh A., Kaur S., Thakur H., Rashi, Kashyap S., Lyudmila A., Mudgal G. (2025). Unveiling the transformative power of smart cellulosic nanomaterials: Revisiting potential promises to sustainable future. Functionalized Cellulose Materials: Sustainable Manufacturing and Applications.

[97] Daneshvar H., Ghordoei Milan K., Sadr A., Sedighy S.H., Malekie S., Mosayebi A. (2021). Multilayer radiation shield for satellite electronic components protection. Sci. Rep..

[98] Rosh-Gorsky A., Coon A., Beck D., D’Onofrio R., Binney Q., Queen I., Barney A., Longton R., Long A.C., Gouker P. (2024). 3D printing of composite radiation shielding for broad spectrum protection of electronic systems. Adv. Mater..

[99] Tulej M., Meyer S., Lüthi M., Lasi D., Galli A., Piazza D., Desorgher L., Reggiani D., Hajdas W., Karlsson S. (2016). Experimental investigation of the radiation shielding efficiency of a MCP detector in the radiation environment near Jupiter’s moon Europa. Nucl. Instrum. Methods Phys. Res. B.

[100] Wang J.Z., Ma J.N., Zhang Q.X., Li Y.C., Jia X.Y., Tian D., Zhu A.W., Qiu J.W. (2020). Optimization design of radioprotection by multilayer materials in Jovian system exploration missions. Spacecr. Environ. Eng..

[101] Cheraghi E. (2022). Fabrication and Evaluation of Polymer Nanocomposites for Space Radiation Shielding Application. Ph.D. Thesis.

[102] Heo Y.J., Park J.K. (2020). A study on the non-toxic compound-based multi-layered radiation shielding sheet and improvement of properties. J. Korean Soc. Radiol..

[103] Spieth B.D., Qassim K.S., Pittman R.N., Russell D.A. (1998). Shielding electronics behind composite structures. IEEE Trans. Nucl. Sci..

[104] Cherng M., Jun I., Jordan T. (2007). Optimum shielding in Jovian radiation environment. Nucl. Instrum. Methods Phys. Res. A.

[105] Vilkov F.E., Lozovan A.A., Bazhanov A.V., Kasitsyn A.N., Schekoturov O.E., Solovev M.K. (2017). Investigation of the radiation-protective properties of a highly filled liquid glass material. J. Surf. Investig. X-Ray Synchrotron Neutron Tech..

[106] DeVanzo M., Hayes R.B. (2020). Ionizing radiation shielding properties of metal oxide impregnated conformal coatings. Radiat. Phys. Chem..

[107] Zhao H.R., Wang J.Q., Chen M.X., Yang T., He C.F., Du H. (2021). Radiation-hardened packaging reinforcement: Design of electron radiation shielding. Space Electron. Technol..

[108] Cordella F., Cappelli M., Ciotti M., Claps G., De Leo V., Mazzotta C., Pacella D., Tamburrino A., Panza F. (2024). Genetic algorithm for multilayer shield optimization with a custom parallel computing architecture. Eur. Phys. J. Plus.

[109] Fetzer A., Anger M., Oleynik P., Praks J. (2024). Total ionising dose multilayer shielding optimisation for nanosatellites on geostationary transfer orbit. Adv. Space Res..

[110] Emmanuel A., Raghavan J., Harris R., Ferguson P. (2014). A comparison of radiation shielding effectiveness of materials for highly elliptical orbits. Adv. Space Res..

[111] Emmanuel A., Raghavan J. (2015). Influence of structure on radiation shielding effectiveness of graphite fiber reinforced polyethylene composite. Adv. Space Res..

[112] Gohel A., Makwana R. (2022). Multi-layered shielding materials for high energy space radiation. Radiat. Phys. Chem..

[113] Han Z.W., Song K.F., Liu S.J., Guo Q.F., Ding G.X., He L.P., Li C.W., Zhang H.J., Liu Y., Chen B. (2023). Lightweight omnidirectional radiation protection for a photon-counting imaging system in space applications. Appl. Sci..

[114] Srinivasan K., Samuel E.J.J. (2017). Evaluation of radiation shielding properties of the polyvinyl alcohol/iron oxide polymer composite. J. Med. Phys..

[115] Her S.C., Su W.B. (2015). Interfacial fracture toughness of multilayer composite structures. Strength Mater..

[116] Zhang L., Zhang X., Song J., Zheng H. (2020). Thermo-induced curvature and interlayer shear stress analysis of MEMS double-layer structure. Contin. Mech. Thermodyn..

[117] Lao Y., Hu S., Shi Y., Deng Y., Wang F., Du H., Zhang H., Wang Y. (2017). Asymmetric interaction of point defects and heterophase interfaces in ZrN/TaN multilayered nanofilms. Sci. Rep..

[118] Pang W., Liu A., Yang K., Chen R., Feng X. (2024). The effect of interface structures on deformation behavior of Cu/Ni multilayer by molecular dynamics. J. Mater..

[119] Wang M., Beyerlein I.J., Zhang J., Han W.Z. (2018). Defect-interface interactions in irradiated Cu/Ag nanocomposites. Acta Mater..

[120] Ferrone K., Willis C., Guan F., Ma J., Peterson L., Kry S. (2023). A review of magnetic shielding technology for space radiation. Radiation.

[121] Mahalingam S., Kang S.G., Kwon D.S., Hossain N., Kim H.K., Manoharan A.K., Bakthavatchalam S., Kim J. (2025). Flexible and lightweight radiation shielding sponges consisting of sulfated tungsten oxide and bismuth halide composites. J. Ind. Eng. Chem..

[122] Leduc R., Ibrahimi N., Dienot J.M., Gavrilenko V., Ruscassie R. (2022). Analytical and 3d numerical study of multilayer shielding effectiveness for board level shielding optimization. Electronics.

[123] Han X.L., Yang Y.K., Xu Y.Y., Hong X.X., Tang Z.Q., Zhang H., Liu N., Li M., Wang Z.M., Zheng A.P. (2024). 3D micro-nano printing technology as a transformative tool apply for microneedle drug delivery. J. Drug Deliv. Sci. Technol..

[124] Cheng X., Jiang C.H., Nagar M., Thakur P., Wan F., Thakur A. (2024). Electromagnetic interference shielding effectiveness of conformal structures based on near-field scanning: A review. Measurement.

[125] Ardiansyah A., Heryanto H., Tahir D. (2024). Textile Shields: Current Bibliometric Analysis of Fabric Integration for EMI Shielding and Prospects for Future Research. Radiat. Phys. Chem..

